# Trial-ready external controls for gene therapy: The MATCH cohort in maple syrup urine disease

**DOI:** 10.1016/j.xcrm.2026.102799

**Published:** 2026-05-12

**Authors:** Karlla W. Brigatti, Ashlin Rodrigues, Erin Sweigert, Joelle Williamson, Alanna Koehler, Grace Loudon Meier, Laura E. Poskitt, Vincent J. Carson, Donna Robinson, Kevin A. Strauss

**Affiliations:** 1 Clinic for Special Children, Gordonville, PA 17529, USA; 2 Plowshare Therapies, Lancaster, PA 17603, USA

**Keywords:** clinical trials, estimand, external control, gene therapy, maple syrup urine disease

## Abstract

Maple syrup urine disease (MSUD) is a life-threatening metabolic disorder for which randomized trials are infeasible. We present the MSUD Age-matched Standard Treatment Cohort (MATCH), a prospective natural history study of 11 infants with classic MSUD followed from neonatal diagnosis to liver transplantation. Aligned with Food and Drug Administration (FDA) guidance and International Council for Harmonisation (ICH) E9(R1), MATCH applies prespecified eligibility criteria, fixed visit cadence, adjudicated outcomes, and explicit handling of intercurrent events. Three of six outcome measures—proportional intact protein equivalent (PIPE), crisis management days (CMDs), and blood alloisoleucine concentration—are prespecified as estimands. Monte Carlo simulations show that a single-arm trial comparing 11 treated participants to MATCH controls achieves ≥90% power (*p* ≤ 0.025) to detect 64% fewer CMDs (3.0% vs. 8.4%), a 57% increase in PIPE (19.3% vs. 12.3%), and a 36% reduction in alloisoleucine (117 vs. 183 μM). MATCH demonstrates how protocolized natural history data serve as regulatory-grade external controls for single-arm trials.

## Highlights

• MATCH establishes a trial-ready external control for MSUD gene therapy
• Three endpoints prespecified as estimands under ICH E9(R1)
• Monte Carlo simulations achieve ≥90% power at α = 0.025
• Blood alloisoleucine positioned as a pharmacodynamic biomarker

## Graphical abstract

**Figure:**
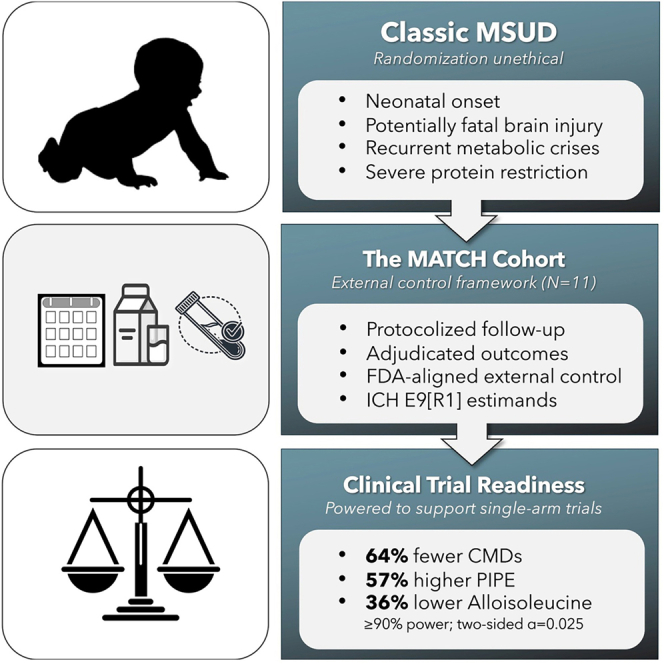

## Teaser

Brigatti et al. develop MATCH, a prospective natural history cohort designed as a regulatory-ready external control for gene therapy trials in maple syrup urine disease. Using prespecified estimands and Monte Carlo simulations, the authors demonstrate how protocolized data can serve as quantitative evidence supporting ethically defensible single-arm trials.

## Introduction

Neonatal-onset (classic) maple syrup urine disease (MSUD; OMIM# 248600) is among the most dangerous inborn errors of metabolism, marked by episodic intoxication, severe dietary constraints, and progressive brain damage.^1^ It is caused by biallelic pathogenic variants in *BCKDHA*, *BCKDHB*, or *DBT*, which encode subunits of the mitochondrial branched-chain 2-ketoacid dehydrogenase complex (BCKDH).^2^ BCKDH is abundant in skeletal muscle, brain, liver, kidney, and heart,^3^ where it decarboxylates 2-ketoacid derivatives of the branched-chain amino acids (BCAAs): leucine, isoleucine, and valine.

Deficiency of BCKDH leads to neurotoxic accumulation of BCAAs and their ketoacids (BCKAs), especially during catabolic states.^4^ Without early detection and expert management, newborns with classic MSUD become comatose within days and can die from brain herniation or central respiratory arrest.^5^ For those who survive, strict dietary demands and the pervasive threat of crisis come to dominate daily decisions and routines,^6^^,^^7^ wearing families down and casting a shadow over their lives.^8^^,^^9^^,^^10^^,^^11^^,^^12^^,^^13^

Allogeneic liver transplantation improves protein tolerance and largely prevents metabolic crises,^14^^,^^15^ but transplant complications are serious and sometimes fatal.^16^ Moreover, neither diet nor transplantation restores critical functions of leucine transamination in the human brain,^17^^,^^18^^,^^19^ where it supplies roughly 50% of the nitrogen required for cerebral glutamate synthesis.^19^^,^^20^ Persistent neurochemical abnormalities in transplanted patients underscore the clinical relevance of this pathway and may contribute to ongoing neuropsychiatric morbidity.^21^^,^^22^ Thus, patients with MSUD need a safer therapy that can be administered early in life to restore BCKDH activity in multiple organs, especially the brain.^3^^,^^23^^,^^24^

Gene-based therapies may offer that possibility.^25^^,^^26^^,^^27^^,^^28^ However, evaluating new treatments with randomized clinical trials is potentially unethical and often infeasible in rare, life-threatening pediatric diseases with a well-defined and consequential natural history.^29^^,^^30^ In this context, natural history and real-world data are increasingly used to support therapeutic development, yet such data are often fragmented or poorly aligned with regulatory requirements.^31^^,^^32^ To address this problem, we previously described the clinical course of MSUD among 184 patients (96% classic), representing 3,512 patient-years and 13,589 blood samples.^1^ That study specified key outcomes and disease biomarkers, inspiring the prospective protocol now used to monitor all patients with MSUD at the Clinic for Special Children (CSC; Gordonville, PA).

Building on this foundation, we used uniform and protocolized longitudinal tracking to minimize the gaps and biases inherent in retrospective and cross-sectional designs.^33^ The result is the MSUD Age-matched Standard Treatment Cohort (MATCH), a prospective natural history cohort deliberately structured to serve as a comparator arm for future interventional trials. MATCH enrollment criteria and endpoint definitions align with U.S. Food and Drug Administration (FDA) draft guidance on externally controlled trials.^34^ In accordance with the International Council for Harmonisation (ICH) E9(R1) addendum, outcomes were framed as estimands to enable rigorous statistical comparison with future treatment cohorts.^35^^,^^36^ Here, we demonstrate a generalizable approach for efficiently converting natural history data into a rigorous, regulatory-aligned external control framework to support single-arm pediatric trials.

## Results

### Outcome measures

Among six clinically relevant outcome measures (Table S1), three were prespecified as estimands for statistical simulation (Table S2; see STAR Methods).^35^^,^^36^ Crisis management days (CMDs) were defined as days of illness requiring complete dietary leucine restriction, whether managed at home or in the hospital. In the absence of such intervention, metabolic crises in classic MSUD are likely to progress to fatal cerebral edema.^5^^,^^37^^,^^38^ Proportional intact protein equivalent (PIPE) quantified the proportion of total ingested protein derived from intact (natural) sources versus BCAA-free formula, where they sum to 100%. A related measure was weight-adjusted leucine tolerance (in mg per kg per day) under stable metabolic control. Among established MSUD biomarkers,^39^ the blood alloisoleucine concentration—comparable across plasma, serum, and dried blood spot matrices^1^^,^^40^—provided the most reliable pathognomonic indicator of BCKDH deficiency,^41^^,^^42^ making it well suited for estimand analysis and regulatory decisions.^35^

### Cohort characteristics

Sixteen high-risk neonates born between 2019 and 2023 underwent targeted genetic testing on the first day of life based on parental carrier status; one additional infant had a positive newborn screen on day of life 8. All 16 were homozygous for an ancestral *BCKDHA* c.1312T>A allele causing classic MSUD.^1^^,^^43^ Five children were excluded because of a confounding condition (*n* = 1; spinal muscular atrophy [SMA]) or insufficient follow-up at the CSC (*n* = 4). Case identification reflected community-based carrier screening practices rather than disease severity.

**Figure 1:**
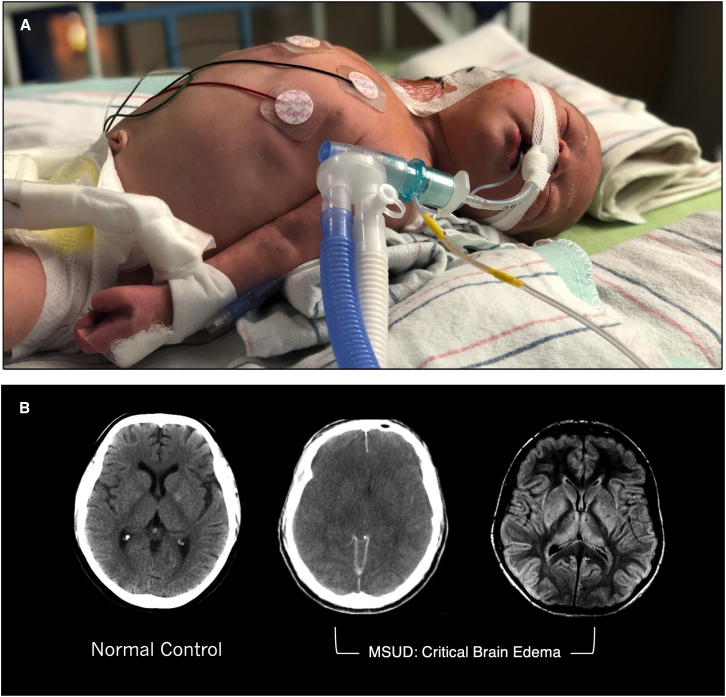
Perinatal metabolic intoxication (A) Opisthotonic posturing and central respiratory failure caused by neurochemical intoxication in MSUD; one of 11 children in the MATCH cohort experienced metabolic encephalopathy shortly after birth and was hospitalized for 18 days. (B) Metabolic intoxication is marked by severe brain swelling (computed tomography, middle) resulting from cytotoxic edema (MRI fluid-attenuated inversion recovery, right). For the one MATCH participant who died after a liver transplant, brain herniation from cerebral edema was the proximate cause of death. See also Table S5. Photo used with permission.

Among the 11 infants included in MATCH (Table 1), three required hospitalization for perinatal metabolic intoxication lasting 5, 10, and 18 days, including one case of severe metabolic encephalopathy (Figure 1). The remaining eight neonates initiated dietary therapy and monitoring within two postnatal days and transitioned safely to home management. As of August 2025, median cohort age was 24.4 months (range, 13.7–39.3). Participants were evaluated in clinic at a mean interval of 29 days (range, 13–57) and underwent quantitative amino acid (AAQ) testing every 5 days on average (range, 3–9).

**Table 1:** Cohort characteristics and measures of metabolic illness (*N* = 11)

	Mean (SD)	Median (IQR)	Range
**General information**			

Gestational age, weeks	39.4 (1.6)	39.9 (38.1–40.4)	37.0–41.1
Birth weight, kg	3.3 (0.5)	3.2 (3.0–3.7)	2.5-4.1
Age at confirmed diagnosis, days	1.7 (2.1)	1.0 (1.0–1.0)	1.0–8.0
Duration of follow up, months	24.6 (7.7)	24.4 (19.1–28.0)	13.7–39.3

**Clinical endpoints**			

Total number of “crisis management days”	62.6 (30.2)	66 (38–78)	8–117
Crisis management days per patient per year	30.5 (15.0)	29 (19–38)	7–54
Proportion of life days in crisis management, %	8.3 (4.1)	8.1 (5.1–10.5)	1.9–14.8
Total number of hospitalizations	2.6 (2.2)	2 (1–4)	0–8
Age when hospitalized, months	11.3 (7.7)	12.4 (3.5–17.6)	0–24.5
Hospital days per patient per year	4.2 (5.3)	1.7 (0.9–5.8)	0–18
Duration of hospital stay, days	3.2 (3.4)	2 (1–4)	1–18
Age at liver transplantation, months^a^	24.6 (7.7)	24.4 (19.1–28.0)	13.7–39.3

Abbreviations are as follows*:* IQR, 25th to 75th interquartile range; SD, one standard deviation.

a All 11 patients received an allogeneic liver transplant.

### Growth and development

All 11 children demonstrated appropriate growth throughout follow-up (Figures S1A and S1B) and achieved independent sitting and walking within 1st–99th percentile reference windows established by the World Health Organization Multicentre Growth Reference Study (WHO-MGRS; Figures S1C and S1D).^44^ Ages of independent sitting (*p* = 0.953) and walking (*p* = 0.861) did not differ from those of healthy Amish and Mennonite control children (*n* = 18).

### CMDs, liver transplantation, and survival

**Figure 2:**
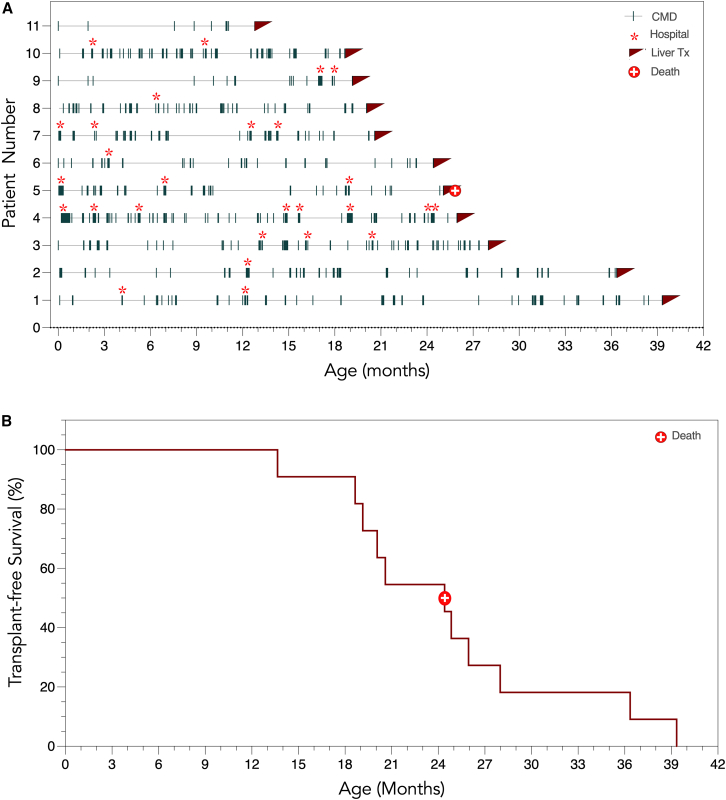
Crisis management days, hospitalizations, and liver transplantation (A) Swimmer plot depicts each MATCH participant on a gray horizontal line. Blue perpendicular hatch marks indicate crisis management days (CMDs), sometimes requiring hospitalization (red asterisks). Triangular symbols denote liver transplants. (B) All 11 children in the cohort underwent liver transplantation at a median age of 24.4 months. One died a week after transplant from post-surgical complications (white “+” in red circle). See also Figure S1 and Table S1.

Across 8,238 aggregate patient-days (22.6 patient-years) of observation, we recorded 689 CMDs (Figure 2A). On average, participants experienced metabolic instability every 12 days (range, every 7–52 days), with CMDs accounting for 8.4 ± 4.1% of lived days (range, 1.9%–14.8%). Episodes managed at home typically required one CMD (range, 1–5) to restore blood leucine concentrations to target levels.

Twenty-nine hospital admissions occurred during follow-up (Figure 2A). Each child experienced a median of two hospitalizations (range, 0–8). Mean plasma leucine concentration at admission was 563 μM (range, 31–2,172), declining by an average of 557 μM per day (range, 228–958) to reach trough values below 100 μM over a median hospital stay of 2 days (range, 0–8). No serious iatrogenic complications were observed.

All 11 participants underwent allogeneic liver transplantation between 13.7 and 39.3 months of age, marking the end of study participation. Kaplan-Meier analysis yielded a median transplant age of 24.4 months and a 100% probability of transplantation by age 39.3 months (Figure 2B). One child died a week after transplantation following an acute graft complication accompanied by hyperleucinemia and hyperammonemia, resulting in fatal cerebral herniation.

### Dietary protein intake and leucine restriction

**Figure 3:**
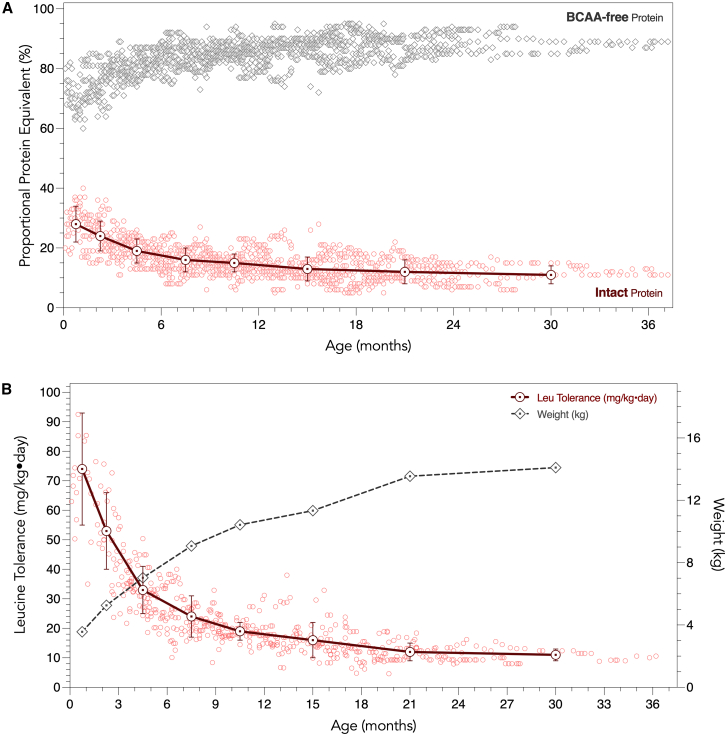
Proportional intact protein intake and leucine tolerance (A) A total of 1,353 diet records were used to calculate proportional intact protein intake (PIPE), representing the percentage of ingested protein from natural (red circles) as compared to BCAA-free (gray diamonds) sources. Mean PIPE decreased from 28% ± 6% in newborns to 12% ± 4% by age 18 months and remained relatively stable thereafter. (B) Leucine tolerance (red circles) showed a similar overall pattern but decreased more markedly than PIPE during the first 18 months of life and was more variable. Weight-adjusted leucine tolerance is related to average growth rate (gray diamonds, right *y* axis). Error bars show one standard deviation. See also Table S3.

PIPE and leucine tolerance were derived from 1,353 diet records. Mean PIPE was 28.4 ± 5.9% (range, 17–40) in newborns and declined to 12.2 ± 3.7% (range, 5–22) by 24 months of age (Table S3, Figure 3A). For power analysis, subject-level mean PIPE values between 12 and 36 months were aggregated, yielding a group mean of 12.3 ± 2.5% (*n* = 617). Leucine tolerance followed a similar age-dependent trajectory but declined more steeply during early infancy (Figure 3B).

### Plasma Biomarkers

**Figure 4:**
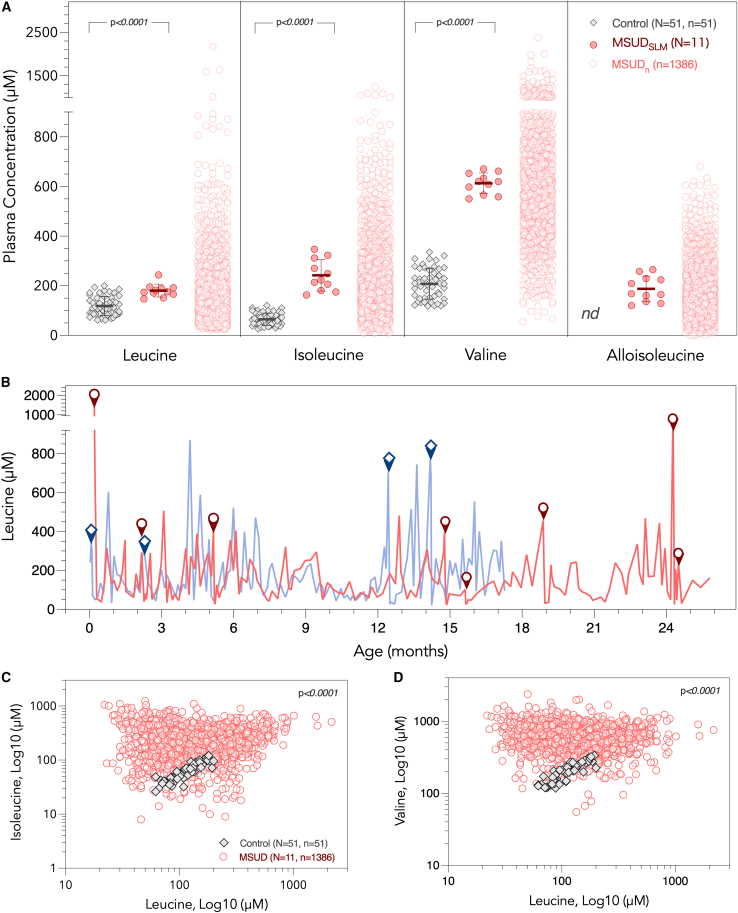
Plasma biomarkers (A) Leucine, isoleucine, and valine concentrations were higher (*p* < 0.0001) and more variable in patients with MSUD than in pediatric control subjects. A total of 1,386 biomarker samples (MSUD_n_; open pink circles) are shown for context only and were used to calculate subject-level means for each of 11 MATCH participants (MSUD_SLM_; filled red circles). To avoid pseudoreplication, 51 control subjects were compared with 11 MSUD_SLM_ values using an unpaired *t* test with Welch’s correction. Alloisoleucine was present in 99.7% of MSUD samples but was not detected (nd) in control samples. (B) Serial leucine concentrations from two MATCH participants illustrate the metabolic volatility of classic MSUD; some excursions required hospitalization (arrowheads). (C and D) Ratios of leucine to isoleucine and valine to leucine are tightly regulated in controls (gray diamonds) but span several orders of magnitude in MSUD (red circles; log10 scales). Error bars in (A) represent mean ± SD. Welch’s *t* tests in (C) and (D) were performed on log10-transformed subject-level mean values. See also Table S4.

Individual blood BCAA concentrations showed limited age dependence and weak intercorrelations (Figure 4A), except for a strong correlation between isoleucine and alloisoleucine (r_s_ = 0.77, *p* ≤ 0.0001). Alloisoleucine was detected in 99.7% of samples from MATCH participants at a mean concentration of 190 ± 120 μM (Table S4) and was undetectable in pediatric control samples. Mean subject-level alloisoleucine concentrations between 12 and 36 months averaged 183 ± 50 μM (*n* = 753) and were used for power simulations.

Intra-individual blood leucine concentrations were highly variable, and peak values did not consistently correspond to hospitalization events (Figure 4B). Compared with pediatric controls, MATCH participants exhibited a 51% higher mean blood leucine concentration (*p* < 0.0001) and substantially greater variability (coefficient of variation, 84% vs. 32%) (Table S4, Figure 4A). Blood valine and isoleucine concentrations were elevated 3-fold and 4-fold, respectively (*p* < 0.0001 for both). Ratios of leucine to isoleucine and valine to leucine were tightly constrained in control children but spanned several orders of magnitude in children with MSUD (Figures 4C and 4D).

### Estimand power analysis

**Figure 5:**
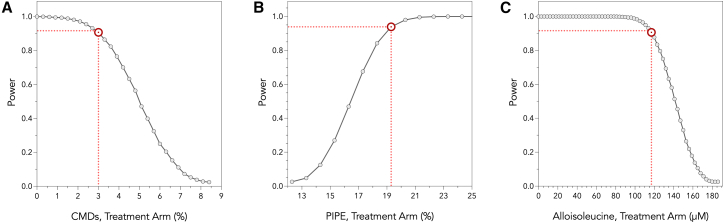
Estimand power analyses (A–C) Power simulations focused on three key estimands: (A) crisis management days (CMDs), (B) proportional intact protein equivalent (PIPE), and (C) blood alloisoleucine concentration. For each estimand, we conducted 10,000 simulations at multiple intervals (gray circles) in a single-arm trial framework comparing 11 MATCH controls to 11 participants treated with an intervention such as gene therapy. Minimum detectable treatment effects with ≥90% power at *p* ≤ 0.025 (red circles with dashed lines) include at least 64% fewer CMDs (3.0% vs. 8.4%; power = 0.907), a 57% or greater increase in PIPE (19.3% vs. 12.3%; power = 0.939), and/or a 36% reduction in blood alloisoleucine concentration (117 vs. 183 μM; power = 0.906). See also Table S2.

Power analyses focused on three prespecified estimands—CMDs, PIPE, and blood alloisoleucine concentration (Table S2). Using subject-level mean values, Monte Carlo simulations evaluated the minimum treatment effects detectable with ≥90% power in a hypothetical single-arm trial comparing 11 treated participants with 11 MATCH controls.^34^ To meet the threshold for substantial evidence of effectiveness at *p* ≤ 0.025,^45^ treated participants would need to experience at least 64% fewer CMDs (3.0% vs. 8.4%; power = 0.907), a ≥57% increase in PIPE (19.3% vs. 12.3%; power = 0.939), and/or a 36% reduction in blood alloisoleucine concentration (117 vs. 183 μM; power = 0.906)(Figure 5). Collectively, these results identify PIPE as the most stable and informative primary efficacy endpoint, with CMDs and alloisoleucine serving as complementary secondary measures.

## Discussion

Despite decades of progress,^1^^,^^46^ MSUD remains a lifelong menace. MATCH participants followed strict diets, attended frequent clinic visits, endured many needle sticks, and faced repeated hospitalizations, yet spent nearly 1 in 10 days under the threat of metabolic crisis. Fragile metabolic control drove all families to seek liver transplantation within the first 40 months of life, and still one child died. These realities underscore the need for therapies that go beyond symptomatic management to address the underlying metabolic defect.

Adeno-associated virus (AAV)-mediated gene replacement is a plausible path forward. It is conceptually similar to liver transplantation—itself a form of gene therapy^47^––but also restores BCKDH activity in native reservoirs of muscle and brain.^3^^,^^22^^,^^28^ Preclinical data support this approach: Pontoizeau et al. rescued *Bckdha−/−* and *Bckdhb−/−* mice using monogenic AAV8 vectors,^25^^,^^26^ and a bidirectional digenic AAV9 vector demonstrated safety and efficacy across two murine models (*Bckdha−/−* and *Bckdhb−/−*) and a large calf with classic MSUD.^28^ Together, these studies establish a strong translational foundation for gene therapy.

MATCH focuses on infants, reflecting a clinical imperative to protect the developing brain.^48^ This strategy is supported by experience in SMA: systemic AAV9 gene replacement therapy (onasemnogene abeparvovec) is relatively safe in newborns and has protected thousands of infants with SMA from permanent neurological damage,^49^^,^^50^ including one Mennonite child born with both classic MSUD and SMA type 1 (CSC case records). The age of MATCH participants reflects the contemporary clinical course of classic MSUD, characterized by neonatal diagnosis, frequent early metabolic instability, and elective liver transplantation in childhood. Study design choices align with regulatory considerations discussed during a February 2025 type B pre-IND meeting with the FDA, including a focus on infant enrollment, patient-relevant endpoints, and dense early follow-up for first-in-human trials.

Randomized, blinded trials remain the evidentiary standard, but in severe pediatric disorders, placebo-controlled designs introduce ethical and practical concerns.^29^^,^^30^ FDA draft guidance recognizes that external controls may be appropriate when the disease course is severe, uniform, well-defined, and unlikely to improve spontaneously.^34^ Classic MSUD meets these criteria. MATCH was therefore deliberately structured to mirror (i.e., “match”) a future treatment cohort with respect to age, genotype, disease severity, observation window, visit cadence, and outcome ascertainment. We prespecified outcomes as estimands under the ICH E9(R1) framework to support quantitative trial design,^35^^,^^36^ and mitigated bias through disciplined eligibility, uniform visit cadence, adjudicated endpoints, and prespecified handling of intercurrent events (Table S2). Collectively, these features satisfy the core evidentiary criteria described in FDA draft guidance for externally controlled trials.^34^

All MATCH participants were homozygous for the *BCKDHA* c.1312T>A ancestral variant. The resulting p.Tyr438Asn substitution produces a functionally null E1α subunit associated with classic MSUD.^1^ This missense variant disrupts BCKDH activity by preventing proper assembly of the catalytic E1 heterotetramer, likely through impaired thiamine pyrophosphate-dependent function.^51^ In cells homozygous for p.Tyr438Asn, both *BCKDHA* (E1α) and *BCKDHB* (E1β) protein levels are markedly reduced, consistent with failure of the E1α–E1β interaction required to form the catalytic core.^51^ Patients harboring this mutation exhibit virtually no residual BCKDH activity (0%–2% of normal)^43^ and manifest the prototypical clinical and biochemical phenotype of classic MSUD.^52^ Prior genotype-phenotype analyses demonstrate that mutations resulting in near-complete loss of E1 catalytic function—whether in *BCKDHA* or *BCKDHB*—produce a convergent phenotype, whereas hypomorphic alleles with residual activity follow more variable clinical trajectories.^2^^,^^39^^,^^53^^,^^54^ Accordingly, although MATCH reflects a single founder genotype, it represents the broader class of severe MSUD cases defined by functional null mutations, which constitute the anticipated target population for early gene replacement trials. Despite this genetic homogeneity, endpoints were defined independently of genotype to enhance transportability, and planned analyses include covariate adjustment for site and care context. Harmonization with international registries will enable multi-center sensitivity analyses and strengthen external validity.

Among candidate outcomes, three emerged as informative estimands: CMDs, PIPE, and blood alloisoleucine concentration. Together, these capture the risk of death or neurologic injury, the lived burden of dietary restriction, and biochemical evidence of BCKDH activity, respectively. Decades of clinical experience and patient feedback indicate that a 64% reduction in CMDs or a 57% increase in PIPE would constitute meaningful benefits.^6^^,^^7^^,^^12^^,^^13^ Either effect, observed in as few as 11 treated participants, could meet our pre-specified statistical threshold (two-sided α = 0.025). We chose this threshold based on FDA guidance, which indicates that a single adequate and well-controlled study intended to support substantial evidence of effectiveness should meet a more stringent statistical standard than the traditional two-trial paradigm (two-sided α = 0.050).^45^ PIPE emerged as the strongest primary efficacy endpoint: stable beyond infancy, clinically meaningful to families, and reliably measured in young children who depend on carefully quantified liquid diets.

Blood alloisoleucine concentration provides a robust pharmacodynamic anchor for therapies aimed at restoring BCKDH function. MATCH strengthens its analytical validity and feasibility by defining variance and longitudinal behavior in a protocolized cohort with structured sampling. Under the FDA’s Biomarker Qualification Program, biomarker use and qualification are context-of-use specific and require evidence supporting both analytical and clinical validation.^55^ In this context, we define the proposed context of use as a pharmacodynamic/response biomarker to support dose selection and early evidence of biologic activity for therapies intended to restore BCKDH function in classic MSUD. Blood alloisoleucine is a robust biomarker in the context of liver transplantation,^1^^,^^40^^,^^41^ and preclinical studies suggest similar utility in the context of gene therapy.^28^ However, to support a claim of surrogate endpoint status as defined by the FDA,^55^ future interventional studies will be required to demonstrate that therapy-induced changes in alloisoleucine are reliable, durable, and quantitatively linked to clinically meaningful outcomes such as CMDs and PIPE.

Growth parameters and early motor milestones were evaluated as exploratory outcomes. As shown in Figure S1, these measures did not distinguish between pediatric control subjects and MATCH participants under standard management. They were therefore deemed unsuitable endpoints. Neuroimaging was considered during study design but not selected as a primary or secondary endpoint. In classic MSUD, acute neurologic injury is driven by episodic metabolic intoxication, whereas magnetic resonance imaging (MRI) abnormalities often lag clinical events. Feasibility is further limited by the ethical and logistical burden of repeated sedation in infants. MRI, and particularly quantitative MR spectroscopy, may serve as exploratory outcome measures in future studies, especially those involving older children or adults.^22^

Beyond MSUD, natural history data for rare diseases are often fragmented and poorly aligned with regulatory needs.^31^^,^^32^ MATCH demonstrates how protocol-driven enrollment, structured longitudinal observation, and prespecified estimands can convert natural history data into a regulatory-ready comparator group for single-arm trials. Although developed for MSUD, this approach is readily adaptable to other rare pediatric disorders in which placebo-controlled trials are impractical or unethical.^30^ Broad sharing of methods and data can facilitate harmonized protocols and enable disparate datasets to be pooled into evidence suitable for regulatory review.^35^ Cohorts like MATCH can serve as engines of trial readiness, accelerating efficient, ethical, and rigorous evaluation of urgently needed therapies.

In summary, MATCH is a trial-ready external control framework aligned with FDA and ICH guidance (Table 2). By combining uniform, protocolized visits, objective endpoints framed as estimands, and simulation-based effect-size thresholds, MATCH provides a credible and ethical comparison standard. MATCH does not establish clinical efficacy of gene therapy but rather defines a disciplined comparator framework against which such efficacy can be rigorously evaluated.

**Table 2:** MATCH versus FDA (2023) guidance and ICH E9(R1) recommendations

FDA/ICH Criterion	MATCH Evidence
Serious, well-defined disease course	classic MSUD: neonatal onset, uniform natural history, life-threatening metabolic crises, progressive brain damage, no spontaneous improvement
Ethics of randomization	placebo or untreated arms unsafe; crises predictably cause neurologic injury/death. ECT offers an ethical comparator
Prospective, protocolized follow-up	11 neonates followed monthly from neonatal diagnosis to transplant; standardized visits, curated endpoints and biomarkers.
Objective, clinically relevant endpoints	three prespecified estimands: (1) CMDs, % of days lived in crisis management; (2) PIPE, % intact protein in diet; and (3) blood alloisoleucine (pathognomonic biomarker); PIPE emerges as primary.
Estimand framework	endpoints defined under ICH E9(R1); intercurrent events prespecified; regulatory-ready statistical analysis plan.
Bias control	uniform eligibility; adjudicated events; standardized definitions; CLIA assays; prespecified handling of missing data
Statistical rigor	Monte Carlo simulations (*N* = 11 vs. 11): ≥90% power at α = 0.025 to detect 64% ↓CMDs, 57% ↑PIPE, 36% ↓alloisoleucine
Transportability and generalizability	founder allele cohort, with phenotype converging across genotypes. Endpoints defined independent of genotype; plans to harmonize with international registries and outside clinical cohorts.
Pediatric acceptability	entire cohort pediatric; aligns with FDA guidance permitting pediatric-first trials when justified
Data provenance and quality	CLIA-certified lab; contemporaneous capture; curated visit cadence; adjudicated outcomes

Abbreviations are as follows*:* CLIA, Clinical Laboratory Improvement Amendments; CMD, crisis management day; ECT, externally controlled trial; FDA, U.S. Food and Drug Administration; ICH, International Council for Harmonisation of Technical Requirements for Pharmaceuticals for Human Use; MATCH, MSUD Age-matched Standard Treatment Cohort; MSUD, maple syrup urine disease; PIPE, proportional intact protein equivalent.

### Limitations of the study

This study is limited by its small size and founder-genotype composition, reflecting the epidemiology of classic MSUD in the Plain communities we serve. Although severe MSUD converges clinically across genotypes, MATCH does not capture milder or intermediate disease. By design, all participants had classic, infantile-onset MSUD, which is clinically and biochemically indistinguishable across complete loss-of-function genotypes in *BCKDHA* (type 1A), *BCKDHB* (type 1B), or *DBT* (type 2).^1^^,^^39^ This represents the population most likely to benefit from early gene therapy. The cohort age distribution reflects current practice favoring early liver transplantation, defining the window for capturing CMDs, PIPE, and alloisoleucine. Finally, while these estimands align with regulatory best practice, their definitive validation will require prospective testing in an interventional trial.

## Resource availability

### Lead contact

Requests for further information and resources should be directed to and will be fulfilled by the lead contact, Kevin A. Strauss (kastrauss@plowsharetherapies.com).

### Materials availability

This study did not generate new unique reagents.

### Data and code availability

• De-identified individual-level data supporting the findings of this study are available from the lead contact (kastrauss@plowsharetherapies.com) upon reasonable request. Requests will be reviewed and responded to within 30 days. A data-sharing agreement may be required to protect participant privacy.
• Simulation code used for Monte Carlo power analyses in R (version 4.5.1) has been uploaded to zenodo (https://doi.org/10.5281/zenodo.19335826) and is available from the lead contact upon request.
• Any additional information required to reanalyze the data reported in this work paper is available from the lead contact upon request.

## Acknowledgments

The authors thank the Clinic for Special Children laboratory team for providing CLIA-certified molecular testing and amino acid analyses in a reliable and prompt manner. The authors are grateful for the outstanding care given to patients with 10.13039/100003936MSUD by the Clinic for Special Children nursing staff. Finally, the authors appreciate the parents of children with MSUD who took part in the MATCH study. These children and their loved ones—past, present, and future—motivate our work. This work was funded in part by charitable donations to the Clinic for Special Children.

## Author contributions

Conceptualization, K.A.S. and K.W.B.; methodology, K.A.S., K.W.B., and A.R.; investigation, K.W.B., E.S., J.W., A.K., G.L.M., L.E.P., V.J.C., D.R., and K.A.S.; visualization: K.A.S. and A.R.; funding acquisition, K.A.S., L.E.P., V.J.C., G.L.M., D.R., and K.W.B.; project administration, K.A.S. and K.W.B.; supervision, K.W.B. and K.A.S.; writing – original draft, K.A.S., A.R., G.L.M., L.E.P., V.J.C., and D.R.; writing – review and editing, K.W.B., K.A.S., A.R., A.K., L.E.P., J.W., and E.S.

## Declaration of interests

K.A.S. is listed as a co-inventor on a patent application filed by the University of Massachusetts Chan Medical School concerning a gene therapy product mentioned in this paper (WO2020210595—AAV-mediated gene therapy for maple syrup urine disease). K.A.S. is the founder of Plowshare Therapies LLC, a biotechnology company in Pennsylvania developing gene therapy for MSUD. Plowshare Therapies provided no direct or indirect funding for this study.

## Declaration of generative AI and AI-assisted technologies in the writing process

During the preparation of this work, K.A.S. used Claude AI (Anthropic) to audit the final manuscript for spelling, verb tense, word counts, and accurate referencing and cross-referencing of tables and figures. After using this service, K.A.S. reviewed the manuscript for verification and takes full responsibility for its final content.

## STAR★Methods

### Key resources table

**Table:** 

REAGENT or RESOURCE	SOURCE	IDENTIFIER
**Biological samples**		

Human plasma samples (venous)	Clinic for Special Children	N/A
Dried blood spot (DBS) samples	Clinic for Special Children	N/A

**Chemicals, peptides, and recombinant proteins**		

Anamix Early Years (MSUD formula)	Nutricia North America	Product Code: 90168
L-Isoleucine (50 g)	Nutricia North America	Product Code: 0170V
L-Valine (50 g)	Nutricia North America	Product Code: 0140I
L-Leucine (50 g)	Nutricia North America	Product Code: 0150L

**Critical commercial assays**		

Quantitative amino acids by HPLC (Agilent system)	Clinic for Special Children (CLIA-certified)	N/A

**Deposited data**		

De-identified individual-level clinical data (MATCH cohort)	This paper; lead contact	N/A

**Software and algorithms**		

GraphPad Prism (version 10.5)	GraphPad Software (San Diego, CA)	RRID:SCR_002798; https://www.graphpad.com
R (version 4.5.1)	R Foundation for Statistical Computing (Vienna, Austria)	RRID:SCR_001905; https://www.r-project.org
Monte Carlo simulation code for power analyses	This paper; lead contact	https://doi.org/10.5281/zenodo.19335826

**Other**		

WHO-MGRS growth reference standards	World Health Organization	https://www.who.int/tools/child-growth-standards/standards/weight-for-age
WHO-MGRS motor milestone windows	World Health Organization	WHO MGRS Group^44^

Abbreviations: N/A, not applicable.

### Experimental model and study participant details

#### Human subjects

The MSUD Age-matched Standard Treatment Cohort (MATCH) represents a convenience population of young children with classic MSUD receiving clinical care at the CSC (Gordonville, PA). All cases diagnosed and managed at CSC between 2019 and 2023 were screened for inclusion. Early diagnosis occurred either through newborn screening or through immediate postnatal testing based on known parental carrier status, reflecting a preemptive population genetics approach rather than disease severity. Participants had a confirmed biochemical and molecular diagnosis of classic MSUD within seven postnatal days. Exclusion criteria were applied uniformly and independent of clinical course.

Children were excluded if they received a substantial portion of their care outside CSC, were unable to adhere to the institution’s treatment and monitoring protocols, or had a confounding medical condition likely to alter clinical course or amino acid homeostasis; exclusions were unrelated to disease severity. The observation window extended from birth through liver transplantation or death, whichever occurred first.

The cohort comprised eight female and three male infants, as assigned by the attending physician at birth. Gestational age, birth weight, age at diagnosis, and duration of follow-up are summarized in Table 1. All participants were otherwise healthy at birth; none had immunodeficiency or clinically significant comorbidities. Participants received standard childhood immunizations in accordance with the CSC treatment protocol (Table S5), with vaccinations held during periods of illness or hyperleucinemia. No participant had received gene therapy, investigational treatment, or liver transplantation prior to enrollment. Dietary management, amino acid monitoring, and crisis management protocols are described in method details.

The study was approved by the Penn Medicine–Lancaster General Hospital Institutional Review Board (Protocol #2008–095-CSC). Written informed consent for research participation was obtained from parents or legal guardians. Parents of the child depicted in Figure 1A signed a photo consent permitting reproduction of the exact image shown.

### Method details

#### Clinical care and follow-up

All participants were followed longitudinally at CSC under a standardized clinical protocol aligned with published consensus standards (Table S5). During the first year of life, patients were typically evaluated by a physician monthly, with visit frequency decreasing to every 2–3 months thereafter. At each clinic visit, clinicians obtained a medical history, performed a physical examination, reviewed dietary records and laboratory results, and documented concomitant treatments and adverse events. Growth and motor development were benchmarked against WHO-MGRS standards,^44^ and growth curves were generated from clinic-recorded measurements.

#### Dietary management

Dietary therapy consisted of intact protein sources (including expressed human milk, commercial infant formulas, and weighed table foods), BCAA-free formula (Nutricia North America, Montreal), and variable quantities of liquid isoleucine, valine, and occasionally leucine (10 mg/mL solutions prepared in distilled water). All dietary components were measured using standardized units (milligrams, grams, milliliters, or ounces) with measuring cups or digital gram scales.

Diet prescriptions were adjusted frequently to (1) maintain plasma leucine concentrations within the reference range observed in healthy pediatric controls (mean 119 ± 38 μM, range 62–200), (2) prevent iatrogenic BCAA deficiencies, and (3) preserve physiologic leucine/isoleucine and valine/leucine ratios (Table S5).

#### Amino acid monitoring

Quantitative amino acids (AAQs) were measured by high-performance liquid chromatography using plasma samples collected during clinic visits or dried blood spot (DBS) samples submitted from home. Families were instructed to submit AAQ samples once or twice weekly between clinic visits.

AAQ results were typically communicated to families within 0.5 h for plasma samples and within 32 h for DBS samples, often accompanied by dietary review and modification. Because reference ranges for BCAAs do not differ between plasma and DBS matrices,^39^^,^^40^ data from both sources were pooled for analysis.

DBS samples were generally collected in the morning before or after the first meal, whereas plasma samples were obtained during daytime clinic visits or inpatient admissions. Blood collection was not timed to feeding or fasting state; therefore, aggregated AAQ data reflect values obtained across a range of physiologic conditions.

#### Management of intercurrent illness

Episodic illnesses were managed at home or in the hospital depending on clinical severity. For non-encephalopathic children with moderate hyperleucinemia, home management consisted of calorie-dense “sick-day” formulas devoid of intact protein and enriched with BCAA-free amino acids. Dietary adjustments were guided by AAQ monitoring every 1–3 days until blood leucine concentrations normalized.

Hospitalization was prompted by encephalopathy, significant catabolic illness, gastrointestinal intolerance, or persistent hyperleucinemia. Inpatient management followed a standardized protocol including BCAA-free parenteral nutrition, intravenous isoleucine and valine supplementation, continuous intravenous insulin, judicious use of hyperosmolar therapy, and laboratory monitoring every six hours (Table S5).^1^^,^^4^^,^^39^

### Quantification and statistical analysis

#### Descriptive and comparative analyses

Statistical analyses were performed using Prism 10.5 (GraphPad). Descriptive statistics are reported as mean ± standard deviation (SD), median with 25th–75th percentile interquartile range (IQR), absolute range, and coefficient of variation (CV). Growth parameters and motor milestones were compared with WHO-MGRS reference standards.^44^ Time-to-event analyses for sitting and walking milestones were compared with those of healthy Amish and Mennonite control children (*n* = 18) using Mantel–Cox log rank tests. The proportional hazards assumption for the Mantel-Cox log rank test was verified by visual inspection of Kaplan-Meier curves. The curves showed similar developmental trajectories between the MATCH cohort and healthy controls, with no sustained divergence, supporting the use of the log rank test for this comparison.

Amino acid concentrations in MATCH participants were compared with pediatric control data using unpaired t-tests with Welch’s correction. Plasma BCAA concentration ratios (leucine/isoleucine and valine/leucine) were log10-transformed before analysis. Associations between variables were evaluated using nonparametric Spearman correlation coefficients (r_s_). For analyses involving repeated measurements, ‘N’ denotes the number of subjects and ‘n’ denotes the number of samples. For comparisons between MATCH participants and pediatric controls in Figure 4, subject-level mean values were used to ensure independence of observations and avoid pseudoreplication arising from unequal sampling frequency.

#### Estimand framework and power simulations

Key outcome measures were framed as estimands in accordance with the ICH E9(R1) addendum, *Estimand and Sensitivity Analysis in Clinical Trials*.^35^ Estimands specify the clinical question of interest and define treatment effects using six components, enabling alignment between outcome definition and statistical inference.^36^

Given the anticipated use of MATCH as an external comparator group for small, single-arm trials, conventional power methods were not appropriate. Power analyses therefore evaluated whether a hypothetical treatment group of 11 participants could achieve ≥90% power to detect clinically meaningful differences relative to MATCH controls and identified the minimum detectable effect size for each outcome. A 1:1 allocation (*N* = 11 per arm) was assumed to reflect feasible recruitment constraints in an ultra-rare pediatric disease and to provide a conservative, realistic comparison framework. Monte Carlo simulations were performed in R (version 4.5.1). Control group means and standard deviations were derived from subject-level averages in MATCH, while treatment group variances were informed by preclinical studies^28^ and comparable clinical cohorts.^1^^,^^15^

Normality assumptions for these simulations were assessed via Shapiro-Wilk testing and found adequate for parametric modeling of subject-level means. To account for parameter uncertainty and potential non-normality in metabolic or rate data, we performed sensitivity analyses by varying hypothesized treatment group standard deviations by ±20%, which confirmed the stability of the reported power estimates and minimum detectable effects.

For a range of plausible treatment means, 10,000 simulated trials were generated assuming normally distributed outcomes and equal allocation (treatment:control). Each simulated dataset was analyzed using Welch’s two-sample *t* test with statistical significance defined as two-sided *p* ≤ 0.025, consistent with FDA recommendations. This threshold was prespecified to reflect FDA guidance, which indicates that a single adequate and well-controlled study intended to support substantial evidence of effectiveness should meet a more stringent statistical standard than the traditional two-trial paradigm (two-sided α = 0.050).^45^ Empirical power was calculated as the proportion of simulations achieving *p* ≤ 0.025, and the smallest treatment mean achieving ≥90% power was recorded. Estimands and their components are summarized in Table S2.

## Supplemental information

Document S1. Figure S1 and Tables S1–S5

Document S2. Article plus supplemental information
