# Supplementary material for: Trial-ready external controls for gene therapy: The MATCH cohort in maple syrup urine disease
Source: Cell Rep Med. 2026 May 12;7(6):102799. doi: 10.1016/j.xcrm.2026.102799 (PMC13293937; doi:10.1016/j.xcrm.2026.102799)
Supplement: Document S1. Figure S1 and Tables S1–S5 [file mmc1.pdf]

Cell Reports Medicine, Volume *7*

Supplemental information

Trial-ready external controls

for gene therapy: The MATCH cohort

in maple syrup urine disease

Karlla W. Brigatti, Ashlin Rodrigues, Erin Sweigert, Joelle Williamson, Alanna Koehler, Grace Loudon Meier, Laura E. Poskitt, Vincent J. Carson, Donna Robinson, and Kevin A. Strauss

Table S1. Six clinically relevant outcome measures to support MSUD gene therapy trials (Related to Figures 2, 3, and 4)

Outcome Measure	Clinical Relevance
**Crisis Management Days**	Each CMD is a proxy for life-threatening metabolic encephalopathy (*5*). Once elevated, neurotoxic concentrations of BCAAs and BCKAs can only be normalized by intensive anabolic therapy (*56*) and/or hemodialysis (*57*), both of which require advanced medical resources and expertise (*39*).
**Proportional Intact Protein Equivalent**	PIPE represents the proportion (%) of total ingested protein from intact (natural) as compared to BCAA-free sources and quantifies how prescription diet differs from normal unrestricted food intake. A PIPE of 100% means 'dietary freedom.'
**Leucine Tolerance**	Leucine tolerance represents the sum of leucine accretion in tissues plus its insensible loss in skin, hair, nails, and excreta *(56, 58)*. It varies directly with growth rate *(1)*.
**Alloisoleucine**	Alloisoleucine is a pathognomonic marker of BCKDH deficiency (*40, 42*). In a large retrospective study of 184 MSUD patients, its blood concentration was 190±117 µM on diet, 4±8 µM after liver transplantation, and undetectable in control subjects (*1*).
**Branched-chain Amino Acids**	Leucine and 2-ketoisocaproic acid are the principal neurotoxins in MSUD and their concentrations in blood correlate with short- and long-term neurological impairments (*23, 59-62*). Following liver transplantation, BCAAs remain modestly elevated but stable in the face of unrestricted daily protein ingestion (*1, 14, 15*).
**BCAA Concentration Ratios**	The intact BCKDH complex maintains concentration relationships among the three BCAAs such that plasma ratios (mol:mol) of leucine to isoleucine (Leu/Ile) and valine to leucine (Val/Leu) are between 1.5 and 2.1 under most conditions (*1*). In contrast, Leu/Ile and Val/Leu ratios vary by orders magnitude in patients with classic MSUD (*1*).

Abbreviations: BCAA, branched-chain amino acid; BCKA, branched-chain 2-ketoacids; BCKDH, branched-chain 2-ketoacid dehydrogenase; CMD, crisis management day; Ile, isoleucine; Leu, leucine; MSUD, maple syrup urine disease; PIPE, proportional intact protein equivalent; Val, valine.

Table S2. Estimand framework for MSUD clinical trials (Related to Figure 5)

	PIPE (%)	CMDs (days/year)	Alloisoleucine (µM)
**Population**	Children from birth to age 24 months with biallelic pathogenic variants in *BCKDHA* or *BCKDHB*, a clinical-biochemical phenotype consistent with MSUD, and no concomitant diagnoses that might confound interpretation of efficacy data.		
**Intervention**	Systemic single-gene or dual-gene replacement or editing of *BCKDHA* and/or *BCKDHB.*		
**Comparison Treatment**	Standard BCAA-restricted dietary therapy paired with inpatient/outpatient crisis management protocols (i.e., current standard of care).		
**Outcome (Meaningful)**	≥50% increase in the proportion of ingested protein from intact ('natural') sources, measured as % of total protein intake.	≥50% reduction in CMDs for management of metabolic instability, counted in days per patient per year.	≥30% reduction of the average alloisoleucine concentration in plasma and/or DBS samples (measured in µM).
**Summary Measure**	Average (SD) age-adjusted PIPE measured between 40 and 52 weeks post-intervention.	Average (SD) frequency of CMDs counted from 12 to 52 weeks post-intervention.	Average (SD) blood alloisoleucine measured between 40 and 52 weeks post-intervention.
**Intercurrent Events**	Patients who die or receive a liver transplant within 40 weeks of intervention are excluded from outcome analysis; i.e. the efficacy cohort includes only children "*while on treatment, while alive*."		

Abbreviations: CMDs, crisis management days; DBS, dried filter paper blood spot; PIPE, proportional intact protein equivalent; SD, one standard deviation.

Table S3. Dietary indices of classic maple syrup urine disease (N=11, n=1353; Related to Figure 3)

	Proportional Intact Protein Equivalent, %					Dietary Leucine Tolerance, mg/kg•day				
Age Range	n[a]	Mean (SD)	Median (IQR)	Range	CV	n[a]	Mean (SD)	Median (IQR)	Range	CV
**Birth to <1.5 mo**	*57*	28.4 (5.9)	30 (24-33)	17-40	21%	*21*	74 (19)	71 (64-85)	35-107	25%
**1.5 to <3 mo**	*83*	24.1 (5.4)	24 (19-28)	15-36	22%	*29*	53 (13)	52 (44-63)	24-77	25%
**3 to <6 mo**	*206*	19.0 (3.7)	19 (17-22)	11-29	20%	*93*	33 (8)	33 (26-37)	18-58	25%
**6 to <9 mo**	*193*	15.9 (4.0)	16 (13-19)	6-27	25%	*85*	24 (7)	22 (19-27)	8-40	28%
**9 to <12 mo**	*197*	14.8 (3.1)	15 (13-16)	6-23	21%	*76*	19 (3)	19 (18-21)	12-30	17%
**12 to <18 mo**	*337*	13.2 (4.4)	12 (10-16)	5-28	33%	*123*	16 (6)	15 (13-18)	5-38	35%
**18 to <24 mo**	*193*	12.2 (3.7)	12 (9-15)	5-22	30%	*85*	12 (3)	11 (9-13)	5-19	26%
**24 to 36 mo**	*87*	11.1 (3.1)	11 (8-14)	6-17	28%	*45*	11 (2)	10 (9-12)	8-16	21%
**12 to 36 mo[b]**	*617*	12.3 (2.5)	13 (11-16)	5-28	32%	*253*	14 (5)	13 (10-16)	5-38	36%

Abbreviations: CV, coefficient of variation; IQR, 25th to 75th interquartile range; mo, months; SD, one standard deviation.

[a] For each participant, individual dietary assessments were spaced at least three days apart. Median sampling frequency was every 5 days (IQR 4-7 days; range: 4-9 days). Leucine tolerance was calculated only on dates that correspond with a clinic visit (i.e., reliable weight measurement), meaning fewer values per age category; **[b]** Anticipating future gene therapy trials, we aggregated ages 12-36 months into a single category (*bottom row*) to perform power analysis for the PIPE estimand.

Table S4. Biomarkers to support clinical trials: MSUD patients (N=11) versus pediatric control subjects (N=51; Related to Figure 4)

	**MSUD** (N=11, n=1729)				**Control** (N=51, n=51)				
	Mean (SD)	Median (IQR)	Range	CV	Mean (SD)	Median (IQR)	Range	CV	p value[b]
**Alloisoleucine, µM**	190 (120)[a]	162 (107-245)	0-893	63%	nd	-		-	na
**Leucine, µM**	180 (151)	137 (85-227)	15-2172	76%	119 (38)	112 (86-151)	62-200	32%	*<0.0001*
**Isoleucine, µM**	246 (187)	198 (126-303)	8-1620	84%	65 (25)	61 (46-84)	26-121	38%	*<0.0001*
**Valine, µM**	609 (246)	573 (447-730)	55-2378	40%	208 (61)	208 (150-263)	118-335	30%	*<0.0001*
**Leu/Ile ratio, mol:mol**	1.14 (1.56)	0.81 (0.42-1.39)	0.01-28.1	136%	1.91 (0.37)	1.83 (1.64-2.13)	1.27-3.41	20%	*<0.0001*
**Val/Leu ratio, mol:mol**	5.95 (6.49)	3.83 (2.25-7.20)	0.27-62.86	109%	1.78 (0.28)	1.77 (1.63-2.00)	1.13-2.49	16%	*<0.0001*

Abbreviations: CV, coefficient of variation; IQR25-75, 25th to 75th interquartile range; N, number of individual subjects; n, number of samples; na, not applicable; nd, not detected; SD, one standard deviation.

[a] For estimand power analysis, we used an average alloisoleucine value of 183±50 µM, which represents the 753 samples obtained between ages 12 and 36 months;
[b] Unpaired, two-tailed t-test with Welch's correction (does not assume equal SDs).

Table S5: Standardized MSUD treatment and monitoring protocol (Clinic for Special Children; Related to Figure 1)

Clinical Context	Treatment Goals	Interventions	Monitoring Principles
**Newborn period**	**1.** Prevent crises<br>**2.** Normal plasma BCAAs<br>**3.** Normal growth	***Asymptomatic Outpatient Management***<br>▪ Only BCAA-free MSUD formula for 24-48 hours<br>▪ Leucine normalizes, start 60-70mg/kg-day leucine from intact source + BCAA-free MSUD formula[a]<br>▪ Supplement 5mg/kg-day L-isoleucine and 20mg/kg-day L-valine; titrate to physiologic ratios<br>▪ Feeding frequency: every 2-3 hours<br>▪ Consider thiamine trial (100-1000 mg).<br>***Symptomatic Infant***<br>▪ Admit for inpatient management	***Plasma Amino Acid Analysis:***<br>▪ Quantitative amino acids (AAQ) at birth or diagnosis<br>▪ Recheck AAQ after 24-48 hour 'washout' with BCAA-free formula only (no intact protein)<br>▪ Then, twice weekly and more frequently as needed
**Outpatient 'Well Day'**	**1.** Normal growth<br>**2.** Normal development<br>**3.** Prevent nutrient deficiencies	***Diet and Monitoring***<br>▪ Age appropriate DRIs for calories and nutrients<br>▪ Total protein: 2-3.5g/ kg-day[b]<br>▪ Intact protein according to leucine tolerance<br>▪ Titrate L-isoleucine (0 -30mg/ kg-day) and L-valine (5-20mg/ kg-day)[c] to physiologic ratios<br>▪ Formula concentration: 0.7-1.0 kcal per mL (20-30 kcal per oz) as tolerated<br>▪ Feeding schedule: *ad lib* from 24-hour volume<br>▪ Standard immunizations[d]<br>▪ Avoid and treat catabolic stressors[e]	***Outpatient Visit Schedule:***<br>▪ Birth to 1 year: every month<br>▪ 1 to 2 years: every 2-4 months<br>▪ 2 years+: every 3-6 months<br>***Plasma Amino Acid Analysis:***<br>▪ Birth to 1 year: twice weekly<br>▪ 12-24 months: weekly<br>▪ 2 years+: every 1-2 weeks<br>▪ Goal: leucine 114 – 190 μmol/L<br>▪ BCAA stoichiometry leucine : isoleucine : valine ratio 2:1:4
**Outpatient 'Sick Day'**	**1.** Reverse catabolism<br>**2.** Promote anabolism<br>**3.** Treat precipitants<br>*4. Hospitalize as indicated*	***Diet and Monitoring***<br>▪ Restrict leucine intake for at least 24 hours<br>▪ BCAA-free protein ≥2-3.5g/ kg-day[b]<br>▪ Caloric intake ≥ resting energy expenditure[g]<br>▪ Formal intake every 1-3 hours, *even overnight*<br>▪ Increase L-isoleucine (30-60mg/kg-day) and L-valine (30-60mg/kg-day) supplementation<br>▪ Low threshold for antimicrobials<br>▪ Antipyretics, antiemetics, and supportive care	***Surveillance Measures***<br>▪ Plasma AAQ daily: target leucine 114-190 μmol/L<br>▪ Office visits to identify treatable illnesses<br>▪ Maintain daily contact with patient/family<br>***Admit to Inpatient for the Following:***<br>▪ Leucine ≥ 760 μmol/L or rapidly increasing<br>▪ Significant encephalopathy or signs of cerebral edema<br>▪ Vomiting, diarrhea, dehydration, or poor oral intake<br>▪ Physical trauma or surgical indication

[a] We use a 24-hour batch consisting of: intact protein source (formula or breastmilk) combined with BCAA-free formula, isoleucine, and valine. Mix additional batch as needed.
[b] Typically 3-3.5g/kg-day for infants < 6 months, 2.5-3g/kg-day for infants 6-12 months, and 1.5-2.5g/kg-day for toddlers.
[c] High relative affinity of leucine for blood-barrier transport compared to valine predisposes to cerebral valine deficiency, which correlates with poor neurocognitive outcomes, making continuous valine supplementation especially important [Muelly 2013]. [f] Higher valine levels (340-680 **μmol**/L) are well-tolerated and may be preferable to avoid cerebral valine deficiency.
[d] Hold immunization when patient is ill or has hyperleucinemia.
[e] Glucocorticoids contraindicated; they promote catabolism and should be avoided except for life-threatening illness.
[g] Minimize physical activity to avoid additional energy expenditure.

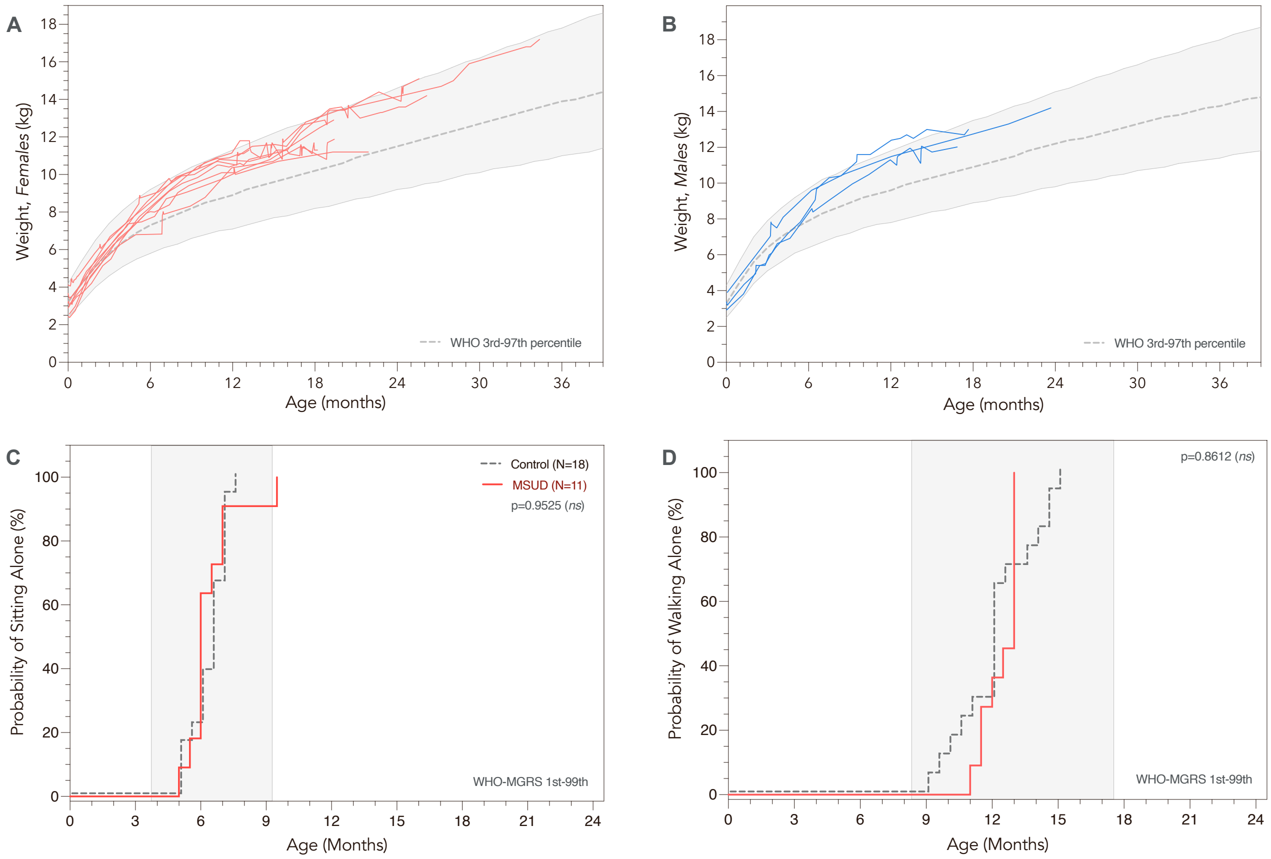

Figure S1. Growth and Early Motor Development. All participants of female (panel **A**, red lines) and male (panel **B**, blue lines) gender grew normally during the first 24 months of life according to World Health Organization (WHO) 3rd-97th percentile reference standards (gray shading). Length and head circumference were also normal (data not shown). All participants achieved independent sitting (panel **C**) and walking (panel **D**) milestones on time, comparable to those of 18 healthy, unrelated children, and within normal windows established by the WHO Multicentre Growth Reference Study (gray shading)[63]. Related to Table 1 and Figure 2.
