# Supplementary material for: Trial-ready external controls for gene therapy: The MATCH cohort in maple syrup urine disease
Source: Cell Rep Med. 2026 May 12;7(6):102799. doi: 10.1016/j.xcrm.2026.102799 (PMC13293937; doi:10.1016/j.xcrm.2026.102799)
Supplement: Document S2. Article plus supplemental information [file mmc2.pdf]

Cell Reports Medicine

Article

Trial-ready external controls for gene therapy: The MATCH cohort in maple syrup urine disease

Graphical abstract

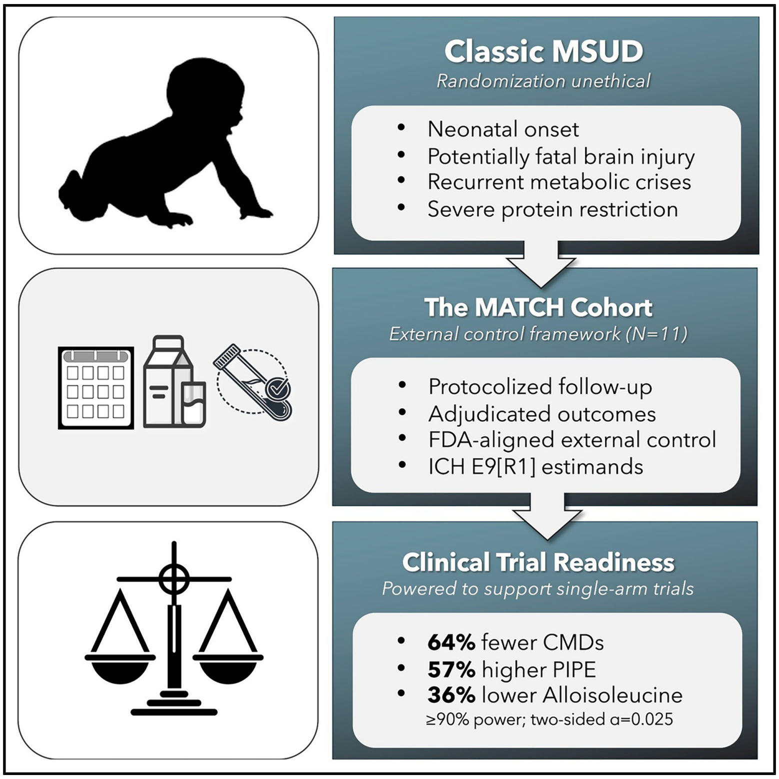

Highlights

- MATCH establishes a trial-ready external control for MSUD gene therapy
- Three endpoints prespecified as estimands under ICH E9(R1)
- Monte Carlo simulations achieve ≥90% power at α = 0.025
- Blood alloisoleucine positioned as a pharmacodynamic biomarker

Authors

Karlla W. Brigatti, Ashlin Rodrigues, Erin Sweigert, ..., Vincent J. Carson, Donna Robinson, Kevin A. Strauss

Correspondence

kastrauss@plowsharetherapies.com

In brief

Brigatti et al. develop MATCH, a prospective natural history cohort designed as a regulatory-ready external control for gene therapy trials in maple syrup urine disease. Using prespecified estimands and Monte Carlo simulations, the authors demonstrate how protocolized data can serve as quantitative evidence supporting ethically defensible single-arm trials.

Brigatti et al., 2026, Cell Reports Medicine 7, 102799
June 16, 2026 © 2026 The Authors. Published by Elsevier Inc.
https://doi.org/10.1016/j.xcrm.2026.102799

Check for updates

CellPress

Cell Reports Medicine

CellPress OPEN ACCESS

Article

Trial-ready external controls for gene therapy: The MATCH cohort in maple syrup urine disease

Karlla W. Brigatti,[1] Ashlin Rodrigues,[1] Erin Sweigert,[1] Joelle Williamson,[1] Alanna Koehler,[1] Grace Loudon Meier,[1] Laura E. Poskitt,[1] Vincent J. Carson,[1] Donna Robinson,[1] and Kevin A. Strauss[1,2,3,*]
[1]Clinic for Special Children, Gordonville, PA 17529, USA
[2]Plowshare Therapies, Lancaster, PA 17603, USA
[3]Lead contact
*Correspondence: kastrauss@plowsharetherapies.com
https://doi.org/10.1016/j.xcrm.2026.102799

SUMMARY

Maple syrup urine disease (MSUD) is a life-threatening metabolic disorder for which randomized trials are infeasible. We present the MSUD Age-matched Standard Treatment Cohort (MATCH), a prospective natural history study of 11 infants with classic MSUD followed from neonatal diagnosis to liver transplantation. Aligned with Food and Drug Administration (FDA) guidance and International Council for Harmonisation (ICH) E9(R1), MATCH applies prespecified eligibility criteria, fixed visit cadence, adjudicated outcomes, and explicit handling of intercurrent events. Three of six outcome measures—proportional intact protein equivalent (PIPE), crisis management days (CMDs), and blood alloisoleucine concentration—are prespecified as estimands. Monte Carlo simulations show that a single-arm trial comparing 11 treated participants to MATCH controls achieves ≥90% power ($p \leq 0.025$) to detect 64% fewer CMDs (3.0% vs. 8.4%), a 57% increase in PIPE (19.3% vs. 12.3%), and a 36% reduction in alloisoleucine (117 vs. 183 μM). MATCH demonstrates how protocolized natural history data serve as regulatory-grade external controls for single-arm trials.

INTRODUCTION

Neonatal-onset (classic) maple syrup urine disease (MSUD; OMIM# 248600) is among the most dangerous inborn errors of metabolism, marked by episodic intoxication, severe dietary constraints, and progressive brain damage.[1] It is caused by biallelic pathogenic variants in *BCKDHA*, *BCKDHB*, or *DBT*, which encode subunits of the mitochondrial branched-chain 2-ketoacid dehydrogenase complex (BCKDH).[2] BCKDH is abundant in skeletal muscle, brain, liver, kidney, and heart,[3] where it decarboxylates 2-ketoacid derivatives of the branched-chain amino acids (BCAAs): leucine, isoleucine, and valine.

Deficiency of BCKDH leads to neurotoxic accumulation of BCAAs and their ketoacids (BCKAs), especially during catabolic states.[4] Without early detection and expert management, newborns with classic MSUD become comatose within days and can die from brain herniation or central respiratory arrest.[5] For those who survive, strict dietary demands and the pervasive threat of crisis come to dominate daily decisions and routines,[6,7] wearing families down and casting a shadow over their lives.[8–13]

Allogeneic liver transplantation improves protein tolerance and largely prevents metabolic crises,[14,15] but transplant complications are serious and sometimes fatal.[16] Moreover, neither diet nor transplantation restores critical functions of leucine transamination in the human brain,[17–19] where it supplies roughly 50% of the nitrogen required for cerebral glutamate synthesis.[19,20] Persistent neurochemical abnormalities in transplanted patients underscore the clinical relevance of this pathway and may contribute to ongoing neuropsychiatric morbidity.[21,22] Thus, patients with MSUD need a safer therapy that can be administered early in life to restore BCKDH activity in multiple organs, especially the brain.[3,23,24]

Gene-based therapies may offer that possibility.[25–28] However, evaluating new treatments with randomized clinical trials is potentially unethical and often infeasible in rare, life-threatening pediatric diseases with a well-defined and consequential natural history.[29,30] In this context, natural history and real-world data are increasingly used to support therapeutic development, yet such data are often fragmented or poorly aligned with regulatory requirements.[31,32] To address this problem, we previously described the clinical course of MSUD among 184 patients (96% classic), representing 3,512 patient-years and 13,589 blood samples.[1] That study specified key outcomes and disease biomarkers, inspiring the prospective protocol now used to monitor all patients with MSUD at the Clinic for Special Children (CSC; Gordonville, PA).

Building on this foundation, we used uniform and protocolized longitudinal tracking to minimize the gaps and biases inherent in retrospective and cross-sectional designs.[33] The result is the MSUD Age-matched Standard Treatment Cohort (MATCH), a prospective natural history cohort deliberately structured to serve as a comparator arm for future interventional trials.

This is an open access article under the CC BY license (http://creativecommons.org/licenses/by/4.0/).

Table 1. Cohort characteristics and measures of metabolic illness (*N* = 11)

	Mean (SD)	Median (IQR)	Range
General information			
Gestational age, weeks	39.4 (1.6)	39.9 (38.1–40.4)	37.0–41.1
Birth weight, kg	3.3 (0.5)	3.2 (3.0–3.7)	2.5-4.1
Age at confirmed diagnosis, days	1.7 (2.1)	1.0 (1.0–1.0)	1.0–8.0
Duration of follow up, months	24.6 (7.7)	24.4 (19.1–28.0)	13.7–39.3
Clinical endpoints			
Total number of "crisis management days"	62.6 (30.2)	66 (38–78)	8–117
Crisis management days per patient per year	30.5 (15.0)	29 (19–38)	7–54
Proportion of life days in crisis management, %	8.3 (4.1)	8.1 (5.1–10.5)	1.9–14.8
Total number of hospitalizations	2.6 (2.2)	2 (1–4)	0–8
Age when hospitalized, months	11.3 (7.7)	12.4 (3.5–17.6)	0–24.5
Hospital days per patient per year	4.2 (5.3)	1.7 (0.9–5.8)	0–18
Duration of hospital stay, days	3.2 (3.4)	2 (1–4)	1–18
Age at liver transplantation, months[a]	24.6 (7.7)	24.4 (19.1–28.0)	13.7–39.3

Abbreviations are as follows: IQR, 25th to 75th interquartile range; SD, one standard deviation.
[a]All 11 patients received an allogeneic liver transplant.

MATCH enrollment criteria and endpoint definitions align with U.S. Food and Drug Administration (FDA) draft guidance on externally controlled trials.[34] In accordance with the International Council for Harmonisation (ICH) E9(R1) addendum, outcomes were framed as estimands to enable rigorous statistical comparison with future treatment cohorts.[35,36] Here, we demonstrate a generalizable approach for efficiently converting natural history data into a rigorous, regulatory-aligned external control framework to support single-arm pediatric trials.

RESULTS

Outcome measures

Among six clinically relevant outcome measures (Table S1), three were prespecified as estimands for statistical simulation (Table S2; see STAR Methods).[35,36] Crisis management days (CMDs) were defined as days of illness requiring complete dietary leucine restriction, whether managed at home or in the hospital. In the absence of such intervention, metabolic crises in classic MSUD are likely to progress to fatal cerebral edema.[5,37,38] Proportional intact protein equivalent (PIPE) quantified the proportion of total ingested protein derived from intact (natural) sources versus BCAA-free formula, where they sum to 100%. A related measure was weight-adjusted leucine tolerance (in mg per kg per day) under stable metabolic control. Among established MSUD biomarkers,[39] the blood alloisoleucine concentration—comparable across plasma, serum, and dried blood spot matrices[1,40]—provided the most reliable pathognomonic indicator of BCKDH deficiency,[41,42] making it well suited for estimand analysis and regulatory decisions.[35]

Cohort characteristics

Sixteen high-risk neonates born between 2019 and 2023 underwent targeted genetic testing on the first day of life based on parental carrier status; one additional infant had a positive newborn screen on day of life 8. All 16 were homozygous for an ancestral *BCKDHA* c.1312T>A allele causing classic MSUD.[1,43] Five children were excluded because of a confounding condition ($n = 1$; spinal muscular atrophy [SMA]) or insufficient follow-up at the CSC ($n = 4$). Case identification reflected community-based carrier screening practices rather than disease severity.

Among the 11 infants included in MATCH (Table 1), three required hospitalization for perinatal metabolic intoxication lasting 5, 10, and 18 days, including one case of severe metabolic encephalopathy (Figure 1). The remaining eight neonates initiated dietary therapy and monitoring within two postnatal days and transitioned safely to home management. As of August 2025, median cohort age was 24.4 months (range, 13.7–39.3). Participants were evaluated in clinic at a mean interval of 29 days (range, 13–57) and underwent quantitative amino acid (AAQ) testing every 5 days on average (range, 3–9).

Growth and development

All 11 children demonstrated appropriate growth throughout follow-up (Figures S1A and S1B) and achieved independent sitting and walking within 1st–99th percentile reference windows established by the World Health Organization Multicentre Growth Reference Study (WHO-MGRS; Figures S1C and S1D).[44] Ages of independent sitting ($p = 0.953$) and walking ($p = 0.861$) did not differ from those of healthy Amish and Mennonite control children ($n = 18$).

CMDs, liver transplantation, and survival

Across 8,238 aggregate patient-days (22.6 patient-years) of observation, we recorded 689 CMDs (Figure 2A). On average, participants experienced metabolic instability every 12 days (range, every 7–52 days), with CMDs accounting for 8.4 ± 4.1% of lived days (range, 1.9%–14.8%). Episodes managed at home typically required one CMD (range, 1–5) to restore blood leucine concentrations to target levels.

Twenty-nine hospital admissions occurred during follow-up (Figure 2A). Each child experienced a median of two hospitalizations (range, 0–8). Mean plasma leucine concentration at admission was 563 μM (range, 31–2,172), declining by an average of 557 μM per day (range, 228–958) to reach trough values below 100 μM over a median hospital stay of 2 days (range, 0–8). No serious iatrogenic complications were observed.

All 11 participants underwent allogeneic liver transplantation between 13.7 and 39.3 months of age, marking the end of study participation. Kaplan-Meier analysis yielded a median transplant age of 24.4 months and a 100% probability of transplantation by age 39.3 months (Figure 2B). One child died a week after

CellPress
OPEN ACCESS

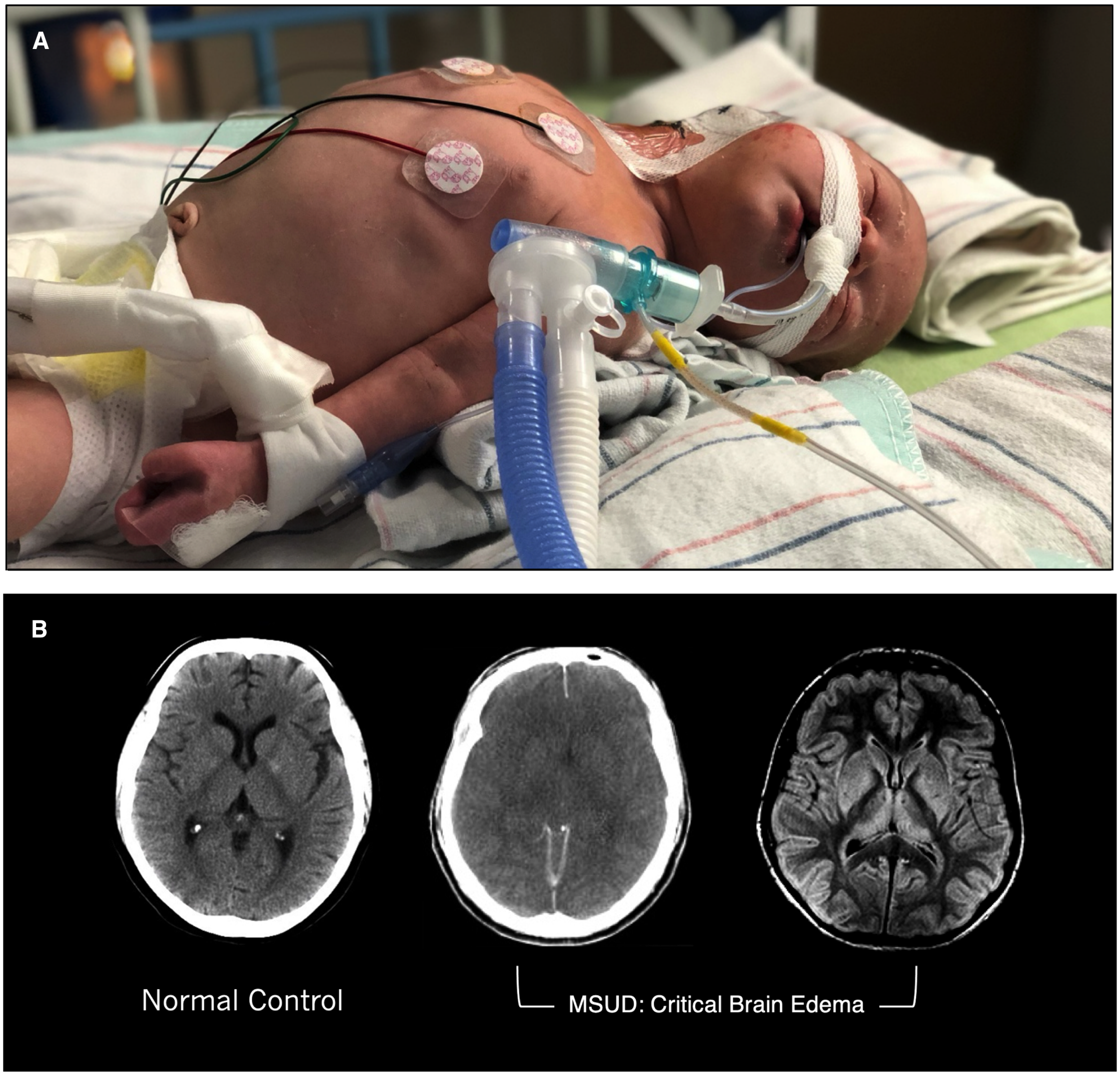

Figure 1. Perinatal metabolic intoxication

(A) Opisthotonic posturing and central respiratory failure caused by neurochemical intoxication in MSUD; one of 11 children in the MATCH cohort experienced metabolic encephalopathy shortly after birth and was hospitalized for 18 days.

(B) Metabolic intoxication is marked by severe brain swelling (computed tomography, middle) resulting from cytotoxic edema (MRI fluid-attenuated inversion recovery, right). For the one MATCH participant who died after a liver transplant, brain herniation from cerebral edema was the proximate cause of death. See also Table S5. Photo used with permission.

transplantation following an acute graft complication accompanied by hyperleucinemia and hyperammonemia, resulting in fatal cerebral herniation.

Dietary protein intake and leucine restriction

PIPE and leucine tolerance were derived from 1,353 diet records. Mean PIPE was 28.4 ± 5.9% (range, 17–40) in newborns and declined to 12.2 ± 3.7% (range, 5–22) by 24 months of age (Table S3, Figure 3A). For power analysis, subject-level mean PIPE values between 12 and 36 months were aggregated, yielding a group mean of 12.3 ± 2.5% (n = 617). Leucine tolerance followed a similar age-dependent trajectory but declined more steeply during early infancy (Figure 3B).

Plasma Biomarkers

Individual blood BCAA concentrations showed limited age dependence and weak intercorrelations (Figure 4A), except for a strong correlation between isoleucine and alloisoleucine (r_s = 0.77, $p \leq 0.0001$). Alloisoleucine was detected in 99.7% of samples from MATCH participants at a mean concentration of 190 ± 120 μM (Table S4) and was undetectable in pediatric control samples. Mean subject-level alloisoleucine concentrations between 12 and 36 months averaged 183 ± 50 μM (n = 753) and were used for power simulations.

Intra-individual blood leucine concentrations were highly variable, and peak values did not consistently correspond to hospitalization events (Figure 4B). Compared with pediatric

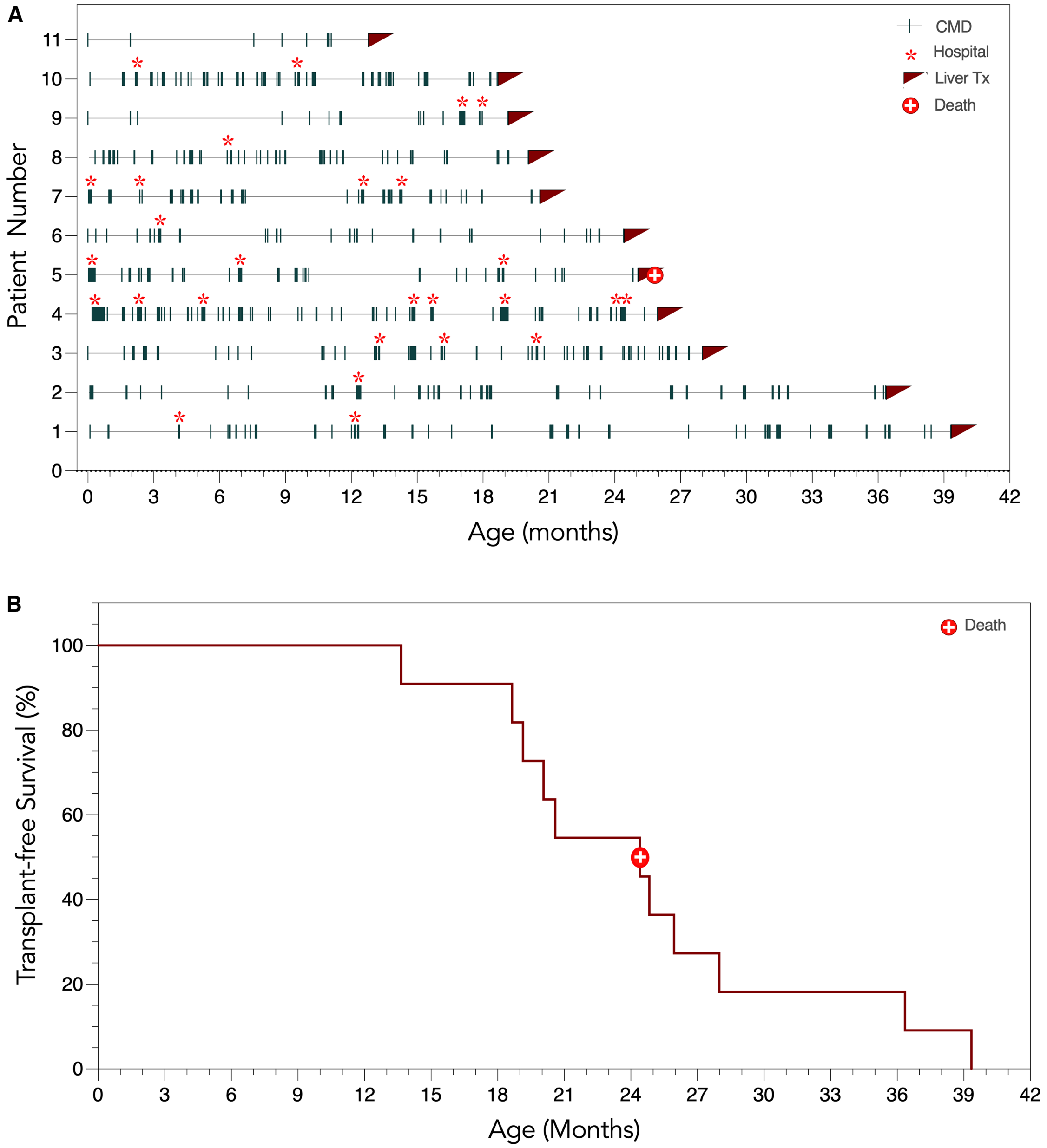

Figure 2. Crisis management days, hospitalizations, and liver transplantation

(A) Swimmer plot depicts each MATCH participant on a gray horizontal line. Blue perpendicular hatch marks indicate crisis management days (CMDs), sometimes requiring hospitalization (red asterisks). Triangular symbols denote liver transplants.

(B) All 11 children in the cohort underwent liver transplantation at a median age of 24.4 months. One died a week after transplant from post-surgical complications (white "+" in red circle). See also Figure S1 and Table S1.

controls, MATCH participants exhibited a 51% higher mean blood leucine concentration ($p < 0.0001$) and substantially greater variability (coefficient of variation, 84% vs. 32%) (Table S4, Figure 4A). Blood valine and isoleucine concentrations were elevated 3-fold and 4-fold, respectively ($p < 0.0001$ for both). Ratios of leucine to isoleucine and valine to leucine were tightly constrained in control children but spanned several orders of magnitude in children with MSUD (Figures 4C and 4D).

Estimand power analysis

Power analyses focused on three prespecified estimands—CMDs, PIPE, and blood alloisoleucine concentration (Table S2). Using subject-level mean values, Monte Carlo simulations evaluated the minimum treatment effects detectable with ≥90% power in a hypothetical single-arm trial comparing 11 treated participants with 11 MATCH controls.[34] To meet the threshold for substantial evidence of effectiveness at $p \leq 0.025$,[45] treated participants would need to experience at least 64% fewer CMDs (3.0% vs.

CellPress
OPEN ACCESS

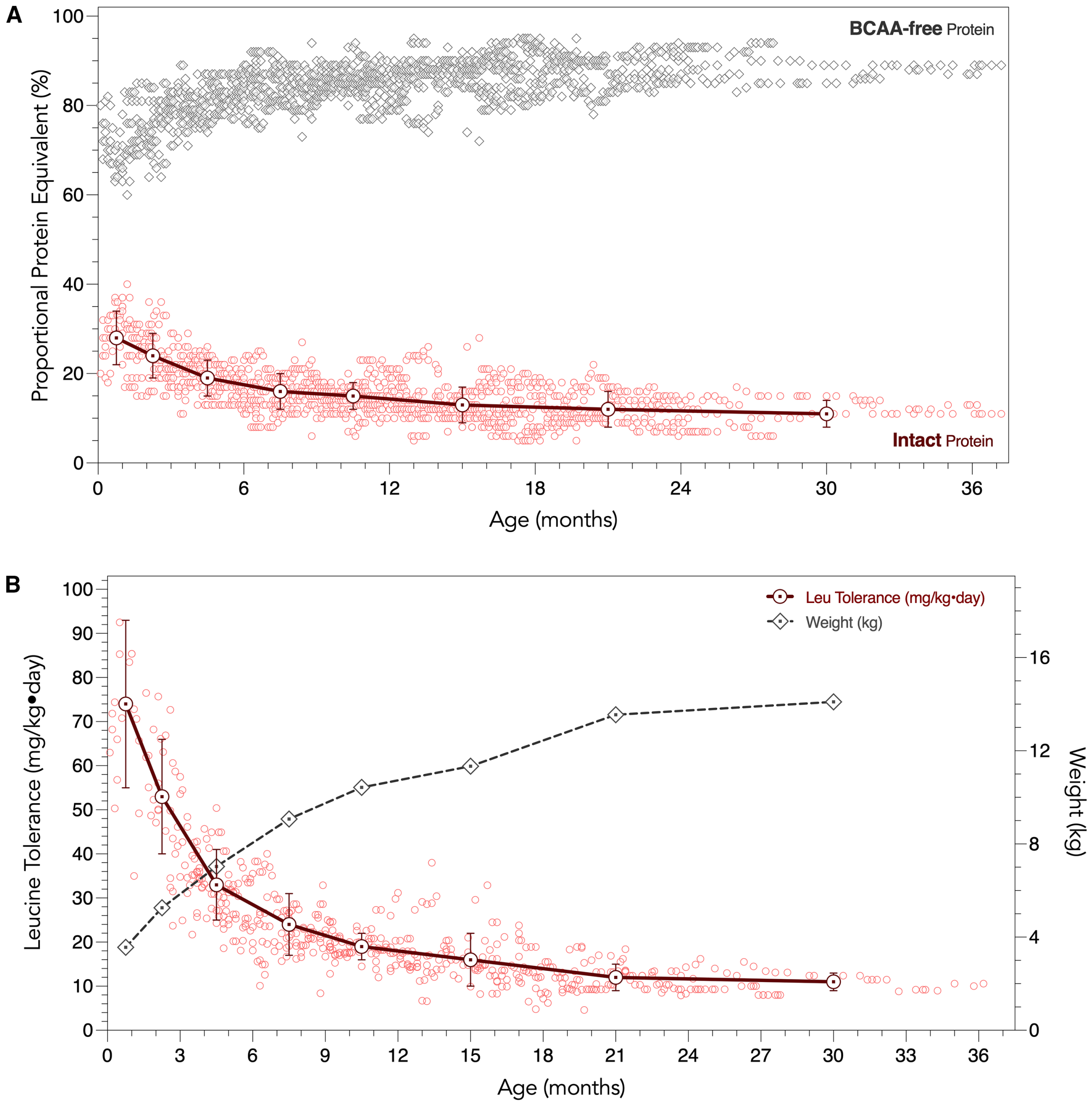

Figure 3. Proportional intact protein intake and leucine tolerance

(A) A total of 1,353 diet records were used to calculate proportional intact protein intake (PIPE), representing the percentage of ingested protein from natural (red circles) as compared to BCAA-free (gray diamonds) sources. Mean PIPE decreased from 28% ± 6% in newborns to 12% ± 4% by age 18 months and remained relatively stable thereafter.

(B) Leucine tolerance (red circles) showed a similar overall pattern but decreased more markedly than PIPE during the first 18 months of life and was more variable. Weight-adjusted leucine tolerance is related to average growth rate (gray diamonds, right *y* axis). Error bars show one standard deviation. See also Table S3.

8.4%; power = 0.907), a ≥57% increase in PIPE (19.3% vs. 12.3%; power = 0.939), and/or a 36% reduction in blood alloisoleucine concentration (117 vs. 183 μM; power = 0.906)(Figure 5). Collectively, these results identify PIPE as the most stable and informative primary efficacy endpoint, with CMDs and alloisoleucine serving as complementary secondary measures.

DISCUSSION

Despite decades of progress,[1,46] MSUD remains a lifelong menace. MATCH participants followed strict diets, attended frequent clinic visits, endured many needle sticks, and faced repeated hospitalizations, yet spent nearly 1 in 10 days under the threat of metabolic crisis. Fragile metabolic control drove all families to seek liver transplantation within the first 40 months of life, and still one child died. These realities underscore the need for therapies that go beyond symptomatic management to address the underlying metabolic defect.

Adeno-associated virus (AAV)-mediated gene replacement is a plausible path forward. It is conceptually similar to liver transplantation—itself a form of gene therapy[47]—but also restores BCKDH activity in native reservoirs of muscle and brain.[3,22,28]

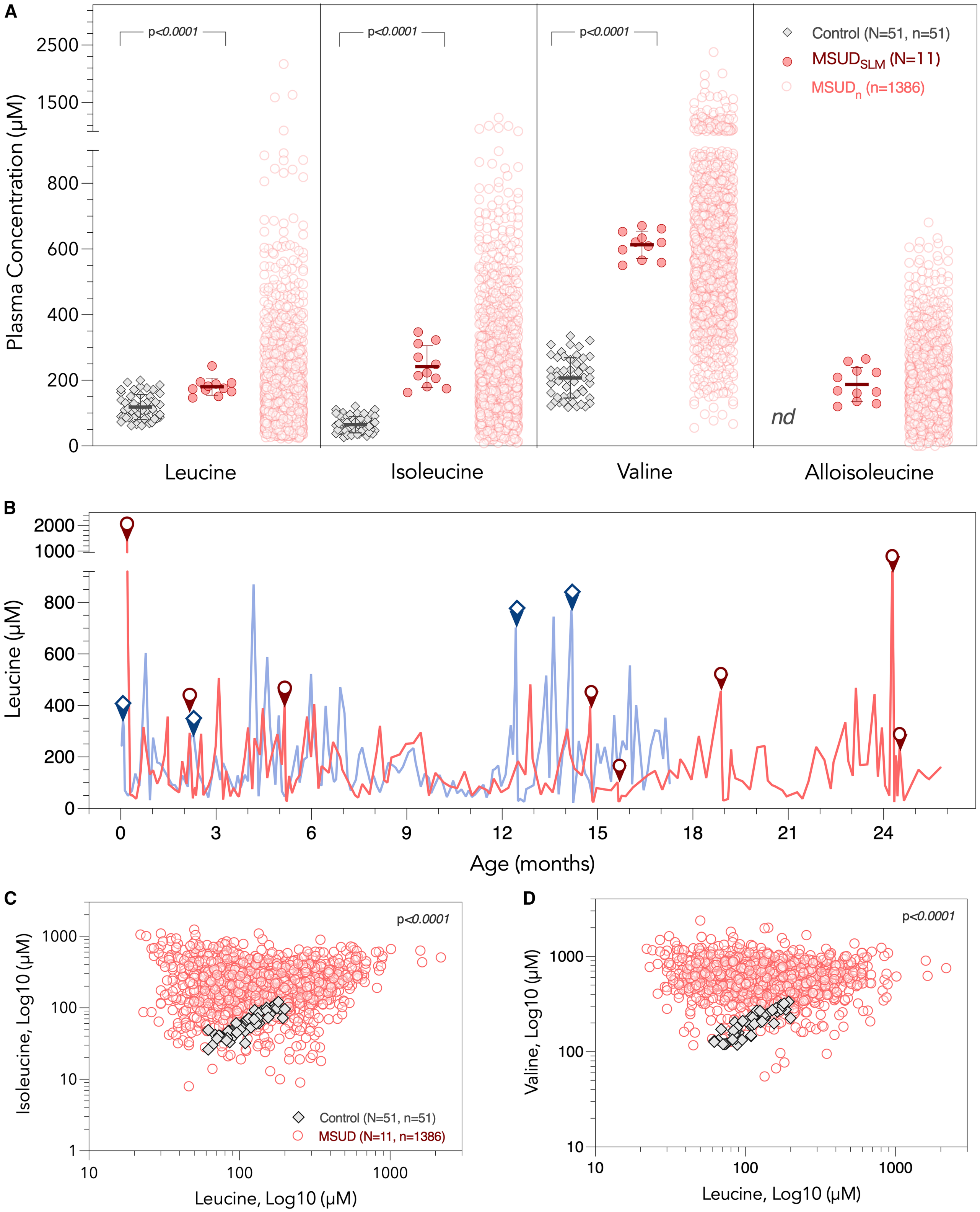

Figure 4. Plasma biomarkers

(A) Leucine, isoleucine, and valine concentrations were higher (p < 0.0001) and more variable in patients with MSUD than in pediatric control subjects. A total of 1,386 biomarker samples (MSUD$_n$; open pink circles) are shown for context only and were used to calculate subject-level means for each of 11 MATCH

(legend continued on next page)

CellPress
OPEN ACCESS

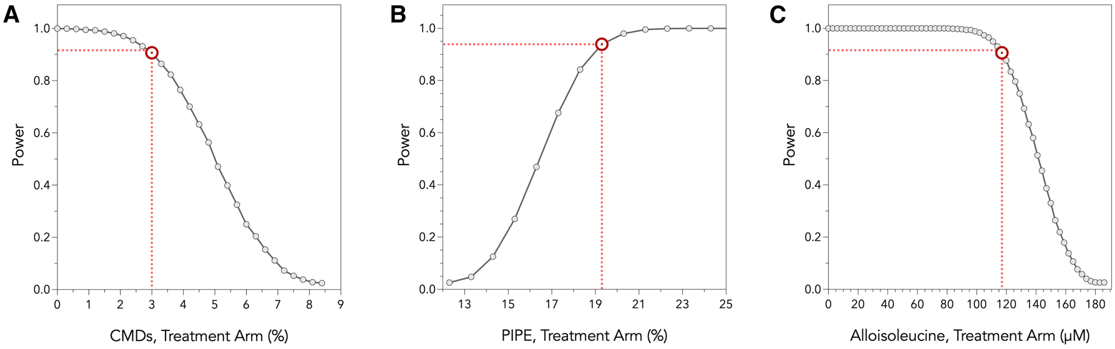

Figure 5. Estimand power analyses

(A–C) Power simulations focused on three key estimands: (A) crisis management days (CMDs), (B) proportional intact protein equivalent (PIPE), and (C) blood alloisoleucine concentration. For each estimand, we conducted 10,000 simulations at multiple intervals (gray circles) in a single-arm trial framework comparing 11 MATCH controls to 11 participants treated with an intervention such as gene therapy. Minimum detectable treatment effects with $\geq$90% power at $p \leq 0.025$ (red circles with dashed lines) include at least 64% fewer CMDs (3.0% vs. 8.4%; power = 0.907), a 57% or greater increase in PIPE (19.3% vs. 12.3%; power = 0.939), and/or a 36% reduction in blood alloisoleucine concentration (117 vs. 183 μM; power = 0.906). See also Table S2.

Preclinical data support this approach: Pontoizeau et al. rescued *Bckdha*−/− and *Bckdhb*−/− mice using monogenic AAV8 vectors,[25,26] and a bidirectional digenic AAV9 vector demonstrated safety and efficacy across two murine models (*Bckdha*−/− and *Bckdhb*−/−) and a large calf with classic MSUD.[28] Together, these studies establish a strong translational foundation for gene therapy.

MATCH focuses on infants, reflecting a clinical imperative to protect the developing brain.[48] This strategy is supported by experience in SMA: systemic AAV9 gene replacement therapy (onasemnogene abeparvovec) is relatively safe in newborns and has protected thousands of infants with SMA from permanent neurological damage,[49,50] including one Mennonite child born with both classic MSUD and SMA type 1 (CSC case records). The age of MATCH participants reflects the contemporary clinical course of classic MSUD, characterized by neonatal diagnosis, frequent early metabolic instability, and elective liver transplantation in childhood. Study design choices align with regulatory considerations discussed during a February 2025 type B pre-IND meeting with the FDA, including a focus on infant enrollment, patient-relevant endpoints, and dense early follow-up for first-in-human trials.

Randomized, blinded trials remain the evidentiary standard, but in severe pediatric disorders, placebo-controlled designs introduce ethical and practical concerns.[29,30] FDA draft guidance recognizes that external controls may be appropriate when the disease course is severe, uniform, well-defined, and unlikely to improve spontaneously.[34] Classic MSUD meets these criteria. MATCH was therefore deliberately structured to mirror (i.e., "match") a future treatment cohort with respect to age, genotype, disease severity, observation window, visit cadence, and outcome ascertainment. We prespecified outcomes as estimands under the ICH E9(R1) framework to support quantitative trial design,[35,36] and mitigated bias through disciplined eligibility, uniform visit cadence, adjudicated endpoints, and prespecified handling of intercurrent events (Table S2). Collectively, these features satisfy the core evidentiary criteria described in FDA draft guidance for externally controlled trials.[34]

All MATCH participants were homozygous for the *BCKDHA* c.1312T>A ancestral variant. The resulting p.Tyr438Asn substitution produces a functionally null E1α subunit associated with classic MSUD.[1] This missense variant disrupts BCKDH activity by preventing proper assembly of the catalytic E1 heterotetramer, likely through impaired thiamine pyrophosphate-dependent function.[51] In cells homozygous for p.Tyr438Asn, both *BCKDHA* (E1α) and *BCKDHB* (E1β) protein levels are markedly reduced, consistent with failure of the E1α–E1β interaction required to form the catalytic core.[51] Patients harboring this mutation exhibit virtually no residual BCKDH activity (0%–2% of normal)[43] and manifest the prototypical clinical and biochemical phenotype of classic MSUD.[52] Prior genotype-phenotype analyses demonstrate that mutations resulting in near-complete loss of E1 catalytic function—whether in *BCKDHA* or *BCKDHB*—produce a convergent phenotype, whereas hypomorphic alleles with residual activity follow more variable clinical trajectories.[2,39,53,54] Accordingly, although MATCH reflects a single founder genotype, it represents the broader class of severe MSUD cases defined by functional null mutations, which constitute the anticipated target population for early gene replacement trials. Despite this genetic homogeneity, endpoints were defined

participants ($MSUD_{SLM}$; filled red circles). To avoid pseudoreplication, 51 control subjects were compared with 11 $MSUD_{SLM}$ values using an unpaired *t* test with Welch's correction. Alloisoleucine was present in 99.7% of MSUD samples but was not detected (nd) in control samples.

(B) Serial leucine concentrations from two MATCH participants illustrate the metabolic volatility of classic MSUD; some excursions required hospitalization (arrowheads).

(C and D) Ratios of leucine to isoleucine and valine to leucine are tightly regulated in controls (gray diamonds) but span several orders of magnitude in MSUD (red circles; log10 scales). Error bars in (A) represent mean ± SD. Welch's *t* tests in (C) and (D) were performed on log10-transformed subject-level mean values. See also Table S4.

Table 2. MATCH versus FDA (2023) guidance and ICH E9(R1) recommendations

FDA/ICH Criterion	MATCH Evidence
Serious, well-defined disease course	classic MSUD: neonatal onset, uniform natural history, life-threatening metabolic crises, progressive brain damage, no spontaneous improvement
Ethics of randomization	placebo or untreated arms unsafe; crises predictably cause neurologic injury/death. ECT offers an ethical comparator
Prospective, protocolized follow-up	11 neonates followed monthly from neonatal diagnosis to transplant; standardized visits, curated endpoints and biomarkers.
Objective, clinically relevant endpoints	three prespecified estimands: (1) CMDs, % of days lived in crisis management; (2) PIPE, % intact protein in diet; and (3) blood alloisoleucine (pathognomonic biomarker); PIPE emerges as primary.
Estimand framework	endpoints defined under ICH E9(R1); intercurrent events prespecified; regulatory-ready statistical analysis plan.
Bias control	uniform eligibility; adjudicated events; standardized definitions; CLIA assays; prespecified handling of missing data
Statistical rigor	Monte Carlo simulations (N = 11 vs. 11): $\geq$90% power at $\alpha = 0.025$ to detect 64% ↓CMDs, 57% ↑PIPE, 36% ↓alloisoleucine
Transportability and generalizability	founder allele cohort, with phenotype converging across genotypes. Endpoints defined independent of genotype; plans to harmonize with international registries and outside clinical cohorts.
Pediatric acceptability	entire cohort pediatric; aligns with FDA guidance permitting pediatric-first trials when justified
Data provenance and quality	CLIA-certified lab; contemporaneous capture; curated visit cadence; adjudicated outcomes

Abbreviations are as follows: CLIA, Clinical Laboratory Improvement Amendments; CMD, crisis management day; ECT, externally controlled trial; FDA, U.S. Food and Drug Administration; ICH, International Council for Harmonisation of Technical Requirements for Pharmaceuticals for Human Use; MATCH, MSUD Age-matched Standard Treatment Cohort; MSUD, maple syrup urine disease; PIPE, proportional intact protein equivalent.

independently of genotype to enhance transportability, and planned analyses include covariate adjustment for site and care context. Harmonization with international registries will enable multi-center sensitivity analyses and strengthen external validity.

Among candidate outcomes, three emerged as informative estimands: CMDs, PIPE, and blood alloisoleucine concentration. Together, these capture the risk of death or neurologic injury, the lived burden of dietary restriction, and biochemical evidence of BCKDH activity, respectively. Decades of clinical experience and patient feedback indicate that a 64% reduction in CMDs or a 57% increase in PIPE would constitute meaningful benefits.[6,7,12,13] Either effect, observed in as few as 11 treated participants, could meet our pre-specified statistical threshold (two-sided $\alpha = 0.025$). We chose this threshold based on FDA guidance, which indicates that a single adequate and well-controlled study intended to support substantial evidence of effectiveness should meet a more stringent statistical standard than the traditional two-trial paradigm (two-sided $\alpha = 0.050$).[45] PIPE emerged as the strongest primary efficacy endpoint: stable beyond infancy, clinically meaningful to families, and reliably measured in young children who depend on carefully quantified liquid diets.

Blood alloisoleucine concentration provides a robust pharmacodynamic anchor for therapies aimed at restoring BCKDH function. MATCH strengthens its analytical validity and feasibility by defining variance and longitudinal behavior in a protocolized cohort with structured sampling. Under the FDA's Biomarker Qualification Program, biomarker use and qualification are context-of-use specific and require evidence supporting both analytical and clinical validation.[55] In this context, we define the proposed context of use as a pharmacodynamic/response biomarker to support dose selection and early evidence of biologic activity for therapies intended to restore BCKDH function in classic MSUD. Blood alloisoleucine is a robust biomarker in the context of liver transplantation,[1,40,41] and preclinical studies suggest similar utility in the context of gene therapy.[28] However, to support a claim of surrogate endpoint status as defined by the FDA,[55] future interventional studies will be required to demonstrate that therapy-induced changes in alloisoleucine are reliable, durable, and quantitatively linked to clinically meaningful outcomes such as CMDs and PIPE.

Growth parameters and early motor milestones were evaluated as exploratory outcomes. As shown in Figure S1, these measures did not distinguish between pediatric control subjects and MATCH participants under standard management. They were therefore deemed unsuitable endpoints. Neuroimaging was considered during study design but not selected as a primary or secondary endpoint. In classic MSUD, acute neurologic injury is driven by episodic metabolic intoxication, whereas magnetic resonance imaging (MRI) abnormalities often lag clinical events. Feasibility is further limited by the ethical and logistical burden of repeated sedation in infants. MRI, and particularly quantitative MR spectroscopy, may serve as exploratory outcome measures in future studies, especially those involving older children or adults.[22]

Beyond MSUD, natural history data for rare diseases are often fragmented and poorly aligned with regulatory needs.[31,32] MATCH demonstrates how protocol-driven enrollment, structured longitudinal observation, and prespecified estimands can convert natural history data into a regulatory-ready comparator group for single-arm trials. Although developed for MSUD, this approach is readily adaptable to other rare pediatric disorders in which placebo-controlled trials are impractical or unethical.[30] Broad sharing of methods and data can facilitate harmonized

CellPress
OPEN ACCESS

protocols and enable disparate datasets to be pooled into evidence suitable for regulatory review.[35] Cohorts like MATCH can serve as engines of trial readiness, accelerating efficient, ethical, and rigorous evaluation of urgently needed therapies.

In summary, MATCH is a trial-ready external control framework aligned with FDA and ICH guidance (Table 2). By combining uniform, protocolized visits, objective endpoints framed as estimands, and simulation-based effect-size thresholds, MATCH provides a credible and ethical comparison standard. MATCH does not establish clinical efficacy of gene therapy but rather defines a disciplined comparator framework against which such efficacy can be rigorously evaluated.

Limitations of the study

This study is limited by its small size and founder-genotype composition, reflecting the epidemiology of classic MSUD in the Plain communities we serve. Although severe MSUD converges clinically across genotypes, MATCH does not capture milder or intermediate disease. By design, all participants had classic, infantile-onset MSUD, which is clinically and biochemically indistinguishable across complete loss-of-function genotypes in *BCKDHA* (type 1A), *BCKDHB* (type 1B), or *DBT* (type 2).[1,39] This represents the population most likely to benefit from early gene therapy. The cohort age distribution reflects current practice favoring early liver transplantation, defining the window for capturing CMDs, PIPE, and alloisoleucine. Finally, while these estimands align with regulatory best practice, their definitive validation will require prospective testing in an interventional trial.

RESOURCE AVAILABILITY

Lead contact

Requests for further information and resources should be directed to and will be fulfilled by the lead contact, Kevin A. Strauss (kastrauss@plowsharetherapies.com).

Materials availability

This study did not generate new unique reagents.

Data and code availability

- De-identified individual-level data supporting the findings of this study are available from the lead contact (kastrauss@plowsharetherapies.com) upon reasonable request. Requests will be reviewed and responded to within 30 days. A data-sharing agreement may be required to protect participant privacy.
- Simulation code used for Monte Carlo power analyses in R (version 4.5.1) has been uploaded to zenodo (https://doi.org/10.5281/zenodo.19335826) and is available from the lead contact upon request.
- Any additional information required to reanalyze the data reported in this work paper is available from the lead contact upon request.

ACKNOWLEDGMENTS

The authors thank the Clinic for Special Children laboratory team for providing CLIA-certified molecular testing and amino acid analyses in a reliable and prompt manner. The authors are grateful for the outstanding care given to patients with MSUD by the Clinic for Special Children nursing staff. Finally, the authors appreciate the parents of children with MSUD who took part in the MATCH study. These children and their loved ones—past, present, and future—motivate our work. This work was funded in part by charitable donations to the Clinic for Special Children.

AUTHOR CONTRIBUTIONS

Conceptualization, K.A.S. and K.W.B.; methodology, K.A.S., K.W.B., and A.R.; investigation, K.W.B., E.S., J.W., A.K., G.L.M., L.E.P., V.J.C., D.R., and K.A.S.; visualization: K.A.S. and A.R.; funding acquisition, K.A.S., L.E.P., V.J.C., G.L.M., D.R., and K.W.B.; project administration, K.A.S. and K.W.B.; supervision, K.W.B. and K.A.S.; writing – original draft, K.A.S., A.R., G.L.M., L.E.P., V.J.C., and D.R.; writing – review and editing, K.W.B., K.A.S., A.R., A.K., L.E.P., J.W., and E.S.

DECLARATION OF INTERESTS

K.A.S. is listed as a co-inventor on a patent application filed by the University of Massachusetts Chan Medical School concerning a gene therapy product mentioned in this paper (WO2020210595—AAV-mediated gene therapy for maple syrup urine disease). K.A.S. is the founder of Plowshare Therapies LLC, a biotechnology company in Pennsylvania developing gene therapy for MSUD. Plowshare Therapies provided no direct or indirect funding for this study.

DECLARATION OF GENERATIVE AI AND AI-ASSISTED TECHNOLOGIES IN THE WRITING PROCESS

During the preparation of this work, K.A.S. used Claude AI (Anthropic) to audit the final manuscript for spelling, verb tense, word counts, and accurate referencing and cross-referencing of tables and figures. After using this service, K.A.S. reviewed the manuscript for verification and takes full responsibility for its final content.

STAR★METHODS

Detailed methods are provided in the online version of this paper and include the following:

- KEY RESOURCES TABLE
- EXPERIMENTAL MODEL AND STUDY PARTICIPANT DETAILS
  - Human subjects
- METHOD DETAILS
  - Clinical care and follow-up
  - Dietary management
  - Amino acid monitoring
  - Management of intercurrent illness
- QUANTIFICATION AND STATISTICAL ANALYSIS
  - Descriptive and comparative analyses
  - Estimand framework and power simulations

SUPPLEMENTAL INFORMATION

Supplemental information can be found online at https://doi.org/10.1016/j.xcrm.2026.102799.

Received: December 22, 2025
Revised: February 28, 2026
Accepted: April 13, 2026
Published: May 12, 2026

REFERENCES

1. Strauss, K.A., Carson, V.J., Soltys, K., Young, M.E., Bowser, L.E., Puffenberger, E.G., Brigatti, K.W., Williams, K.B., Robinson, D.L., Hendrickson, C., et al. (2020). Branched-chain alpha-ketoacid dehydrogenase deficiency (maple syrup urine disease): Treatment, biomarkers, and outcomes. Mol. Genet. Metab. *129*, 193–206. https://doi.org/10.1016/j.ymgme.2020.01.006.
2. Chuang, D.T., and Shih, V.E. (2001). Maple syrup urine disease (branched-chain ketoaciduria). In The Metabolic and Molecular Bases of Inherited

Disease, C.R. Scriver, A.L. Beaudet, D. Valle, and W.S. Sly, eds. (McGraw-Hill), pp. 1971–2006.

3. Suryawan, A., Hawes, J.W., Harris, R.A., Shimomura, Y., Jenkins, A.E., and Hutson, S.M. (1998). A molecular model of human branched-chain amino acid metabolism. Am. J. Clin. Nutr. *68*, 72–81.

4. Strauss, K.A. (2021). Metabolic Crises. In Pediatric Critical Care: Text and Study Guide, S.E. Lucking, F.A. Maffei, R.F. Tamburro, and A. Zaritsky, eds. (Cham: Springer), pp. 1351–1396.

5. Riviello, J.J., Jr., Rezvani, I., DiGeorge, A.M., and Foley, C.M. (1991). Cerebral edema causing death in children with maple syrup urine disease. J. Pediatr. *119*, 42–45.

6. Packman, W., Mehta, I., Rafie, S., Mehta, J., Naldi, M., and Mooney, K.H. (2012). Young adults with MSUD and their transition to adulthood: psychosocial issues. J. Genet. Couns. *21*, 692–703. https://doi.org/10.1007/s10897-012-9490-1.

7. Packman, W., Henderson, S.L., Mehta, I., Ronen, R., Danner, D., Chesterman, B., and Packman, S. (2007). Psychosocial issues in families affected by maple syrup urine disease. J. Genet. Couns. *16*, 799–809. https://doi.org/10.1007/s10897-007-9114-3.

8. Bösch, F., Landolt, M.A., Baumgartner, M.R., Fernandez, S., Forny, P., Gautschi, M., Grünert, S.C., Häberle, J., Horvath, C., Karall, D., et al. (2022). Caregiver burden, and parents' perception of disease severity determine health-related quality of life in paediatric patients with intoxication-type inborn errors of metabolism. Mol. Genet. Metab. Rep. *31*, 100876. https://doi.org/10.1016/j.ymgmr.2022.100876.

9. Bösch, F., Landolt, M.A., Baumgartner, M.R., Zeltner, N., Kölker, S., Gleich, F., Burlina, A., Cazzorla, C., Packman, W., V D Schwartz, I., et al. (2021). Health-related quality of life in paediatric patients with intoxication-type inborn errors of metabolism: Analysis of an international data set. J. Inherit. Metab. Dis. *44*, 215–225. https://doi.org/10.1002/jimd.12301.

10. Fabre, A., Baumstarck, K., Cano, A., Loundou, A., Berbis, J., Chabrol, B., and Auquier, P. (2013). Assessment of quality of life of the children and parents affected by inborn errors of metabolism with restricted diet: preliminary results of a cross-sectional study. Health Qual. Life Outcomes *11*, 158. https://doi.org/10.1186/1477-7525-11-158.

11. Greco, B., Caviglia, S., Martinelli, D., Capitello, T.G., Liccardo, D., De Nictolis, F., Pietrobattista, A., Huemer, M., Piga, S., Olivieri, G., et al. (2023). The impact of liver transplantation on health-related quality of life in (acute) intoxication-type inborn errors of metabolism. J. Inherit. Metab. Dis. *46*, 906–915. https://doi.org/10.1002/jimd.12648.

12. Ouattara, A., Resseguier, N., Cano, A., De Lonlay, P., Arnoux, J.B., Brassier, A., Schiff, M., Pichard, S., Fabre, A., Hoebeke, C., et al. (2023). Individual and Family Determinants for Quality of Life in Parents of Children with Inborn Errors of Metabolism Requiring a Restricted Diet: A Multilevel Analysis Approach. J. Pediatr. *254*, 39–47.e4. https://doi.org/10.1016/j.jpeds.2022.08.060.

13. Ouattara, A., Resseguier, N., Cano, A., De Lonlay, P., Arnoux, J.B., Brassier, A., Schiff, M., Pichard, S., Fabre, A., Hoebeke, C., et al. (2022). Determinants of Quality of Life in Children with Inborn Errors of Metabolism Receiving a Restricted Diet. J. Pediatr. *242*, 192–200.e3. https://doi.org/10.1016/j.jpeds.2021.11.021.

14. Strauss, K.A., Mazariegos, G.V., Sindhi, R., Squires, R., Finegold, D.N., Vockley, G., Robinson, D.L., Hendrickson, C., Virji, M., Cropcho, L., et al. (2006). Elective liver transplantation for the treatment of classical maple syrup urine disease. Am. J. Transplant. *6*, 557–564.

15. Mazariegos, G.V., Morton, D.H., Sindhi, R., Soltys, K., Nayyar, N., Bond, G., Shellmer, D., Shneider, B., Vockley, J., and Strauss, K.A. (2012). Liver transplantation for classical maple syrup urine disease: long-term follow-up in 37 patients and comparative United Network for Organ Sharing experience. J. Pediatr. *160*, 116–121.e1. https://doi.org/10.1016/j.jpeds.2011.06.033.

16. Kohli, R., Cortes, M., Heaton, N.D., and Dhawan, A. (2018). Liver transplantation in children: state of the art and future perspectives. Arch. Dis. Child. *103*, 192–198. https://doi.org/10.1136/archdischild-2015-310023.

17. Yudkoff, M., Daikhin, Y., Grunstein, L., Nissim, I., Stern, J., Pleasure, D., and Nissim, I. (1996). Astrocyte leucine metabolism: significance of branched-chain amino acid transamination. J. Neurochem. *66*, 378–385.

18. Kanamori, K., Ross, B.D., and Kondrat, R.W. (1998). Rate of glutamate synthesis from leucine in rat brain measured in vivo by 15N NMR. J. Neurochem. *70*, 1304–1315. https://doi.org/10.1046/j.1471-4159.1998.70031304.x.

19. McKenna, M.C. (2007). The glutamate-glutamine cycle is not stoichiometric: fates of glutamate in brain. J. Neurosci. Res. *85*, 3347–3358. https://doi.org/10.1002/jnr.21444.

20. Hutson, S.M., Berkich, D., Drown, P., Xu, B., Aschner, M., and LaNoue, K.F. (1998). Role of branched-chain aminotransferase isoenzymes and gabapentin in neurotransmitter metabolism. J. Neurochem. *71*, 863–874.

21. Kuhs, A.C., Ohl, L., Thurston, T., Singh, J., Bhuyan, S., Grandinette, S., Xu, J., Siemsgluess, S.A., Jakher, Y., and Ahrens-Nicklas, R.C. (2025). Contribution of Brain Intrinsic Branched-Chain Amino Acid Metabolism in a Novel Mouse Model of Maple Syrup Urine Disease. J. Inherit. Metab. Dis. *48*, e70003. https://doi.org/10.1002/jimd.70003.

22. Muelly, E.R., Moore, G.J., Bunce, S.C., Mack, J., Bigler, D.C., Morton, D.H., and Strauss, K.A. (2013). Biochemical correlates of neuropsychiatric illness in maple syrup urine disease. J. Clin. Investig. *123*, 1809–1820. https://doi.org/10.1172/JCI67217.

23. Yudkoff, M., Daikhin, Y., Nissim, I., Horyn, O., Luhovyy, B., Luhovyy, B., Lazarow, A., and Nissim, I. (2005). Brain amino acid requirements and toxicity: the example of leucine. J. Nutr. *135*, 1531S–1538S.

24. Yudkoff, M., Daikhin, Y., Nissim, I., Pleasure, D., Stern, J., and Nissim, I. (1994). Inhibition of astrocyte glutamine production by alpha-ketoisocaproic acid. J. Neurochem. *63*, 1508–1515.

25. Pontoizeau, C., Gaborit, C., Tual, N., Simon-Sola, M., Rotaru, I., Benoist, M., Colella, P., Lamazière, A., Brassier, A., Arnoux, J.B., et al. (2024). Successful treatment of severe MSUD in Bckdhb(-/-) mice with neonatal AAV gene therapy. J. Inherit. Metab. Dis. *47*, 41–49. https://doi.org/10.1002/jimd.12604.

26. Pontoizeau, C., Simon-Sola, M., Gaborit, C., Nguyen, V., Rotaru, I., Tual, N., Colella, P., Girard, M., Biferi, M.G., Arnoux, J.B., et al. (2022). Neonatal gene therapy achieves sustained disease rescue of maple syrup urine disease in mice. Nat. Commun. *13*, 3278. https://doi.org/10.1038/s41467-022-30880-w.

27. Greig, J.A., Jennis, M., Dandekar, A., Chorazeczewski, J.K., Smith, M.K., Ashley, S.N., Yan, H., and Wilson, J.M. (2021). Muscle-directed AAV gene therapy rescues the maple syrup urine disease phenotype in a mouse model. Mol. Genet. Metab. *134*, 139–146. https://doi.org/10.1016/j.ymgme.2021.08.003.

28. Wang, J., Poskitt, L.E., Gallagher, J., Puffenberger, E.G., Wynn, R.M., Shishodia, G., Chuang, D.T., Beever, J., Hardin, D.L., Brigatti, K.W., et al. (2025). BCKDHA-BCKDHB digenic gene therapy restores metabolic homeostasis in two mouse models and a calf with classic maple syrup urine disease. Sci. Transl. Med. *17*, eads0539. https://doi.org/10.1126/scitranslmed.ads0539.

29. Millum, J., and Grady, C. (2013). The ethics of placebo-controlled trials: methodological justifications. Contemp. Clin. Trials *36*, 510–514. https://doi.org/10.1016/j.cct.2013.09.003.

30. de Melo-Martin, I., Sondhi, D., and Crystal, R.G. (2011). When ethics constrains clinical research: trial design of control arms in "greater than minimal risk" pediatric trials. Hum. Gene Ther. *22*, 1121–1127. https://doi.org/10.1089/hum.2010.230.

31. Denton, N., Mulberg, A.E., Molloy, M., Charleston, S., Fajgenbaum, D.C., Marsh, E.D., and Howard, P. (2022). Sharing is caring: a call for a new era of rare disease research and development. Orphanet J. Rare Dis. *17*, 389. https://doi.org/10.1186/s13023-022-02529-w.

32. Denton, N., Molloy, M., Charleston, S., Lipset, C., Hirsch, J., Mulberg, A.E., Howard, P., and Marsh, E.D. (2021). Data silos are undermining drug development and failing rare disease patients. Orphanet J. Rare Dis. *16*, 161. https://doi.org/10.1186/s13023-021-01806-4.

33. Talari, K., and Goyal, M. (2020). Retrospective studies - utility and caveats. J. R. Coll. Physicians Edinb. *50*, 398–402. https://doi.org/10.4997/JRCPE.2020.409.

34. FDA (2023). Draft Guidance: Considerations for the Design and Conduct of Externally Controlled Trials for Drug and Biological Products. https://www.fda.gov/drugs/guidance-compliance-regulatory-information/guidances-drugs.

35. Kahan, B.C., Hindley, J., Edwards, M., Cro, S., and Morris, T.P. (2024). The estimands framework: a primer on the ICH E9(R1) addendum. BMJ *384*, e076316. https://doi.org/10.1136/bmj-2023-076316.

36. Troxel, A.B., Gatsonis, C.A., Hogan, J.W., Hubbard, R.A., Hunter, D.J., and Normand, S.L.T. (2026). Statistics in Medicine - What's in an Estimand? N. Engl. J. Med. *394*, 6–9. https://doi.org/10.1056/NEJMp2513633.

37. Menkes, J.H., Hurst, P.L., and Craig, J.M. (1954). A new syndrome: progressive familial infantile cerebral dysfunction associated with an unusual urinary substance. Pediatrics *14*, 462–467.

38. Sulaiman, R.A., Altassan, R., Alshammari, R., Alsayed, M., Sheikh, A.N., Nicolas-Jilwan, M., Al-Owain, M., and Al-Hassnan, Z. (2025). Cerebral edema in maple syrup urine disease: spectrum of clinical presentation and treatment outcomes. Orphanet J. Rare Dis. *20*, 594. https://doi.org/10.1186/s13023-025-04043-1.

39. Strauss, K.A., Puffenberger, E.G., and Morton, D.H. (2009). Maple Syrup Urine Disease. http://www.ncbi.nlm.nih.gov/bookshelf/br.fcgi?book=gene&part=msud.

40. Oglesbee, D., Sanders, K.A., Lacey, J.M., Magera, M.J., Casetta, B., Strauss, K.A., Tortorelli, S., Rinaldo, P., and Matern, D. (2008). Second-tier test for quantification of alloisoleucine and branched-chain amino acids in dried blood spots to improve newborn screening for maple syrup urine disease (MSUD). Clin. Chem. *54*, 542–549. https://doi.org/10.1373/clinchem.2007.098434.

41. Schadewaldt, P., Bodner-Leidecker, A., Hammen, H.W., and Wendel, U. (1999). Significance of L-alloisoleucine in plasma for diagnosis of maple syrup urine disease. Clin. Chem. *45*, 1734–1740.

42. Schadewaldt, P., Dalle-Feste, C., Langenbeck, U., and Wendel, U. (1991). Oral L-alloisoleucine loading studies in healthy subjects and in patients with maple syrup urine disease. Pediatr. Res. *30*, 430–434. https://doi.org/10.1203/00006450-199111000-00007.

43. Nellis, M.M., Kasinski, A., Carlson, M., Allen, R., Schaefer, A.M., Schwartz, E.M., and Danner, D.J. (2003). Relationship of causative genetic mutations in maple syrup urine disease with their clinical expression. Mol. Genet. Metab. *80*, 189–195.

44. WHO Multicentre Growth Reference Study Group (2006). WHO Motor Development Study: windows of achievement for six gross motor development milestones. Acta Paediatr. Suppl. *450*, 86–95. https://doi.org/10.1111/j.1651-2227.2006.tb02379.x.

45. FDA (2019). Demonstrating Substantial Evidence of Effectiveness for Human Drug and Biological Products. https://www.regulations.gov.

46. Strauss, K.A., Puffenberger, E.G., and Morton, D.H. (2012). One community's effort to control genetic disease. Am. J. Public Health *102*, 1300–1306. https://doi.org/10.2105/AJPH.2011.300569.

47. Feier, F.H., Miura, I.K., Fonseca, E.A., Porta, G., Pugliese, R., Porta, A., Schwartz, I.V.D., Margutti, A.V.B., Camelo, J.S., Jr., Yamaguchi, S.N., et al. (2014). Successful domino liver transplantation in maple syrup urine disease using a related living donor. Braz. J. Med. Biol. Res. *47*, 522–526.

48. Govoni, A., Gagliardi, D., Comi, G.P., and Corti, S. (2018). Time Is Motor Neuron: Therapeutic Window and Its Correlation with Pathogenetic Mechanisms in Spinal Muscular Atrophy. Mol. Neurobiol. *55*, 6307–6318. https://doi.org/10.1007/s12035-017-0831-9.

49. Day, J.W., Mendell, J.R., Mercuri, E., Finkel, R.S., Strauss, K.A., Kleyn, A., Tauscher-Wisniewski, S., Tukov, F.F., Reyna, S.P., and Chand, D.H. (2021). Clinical Trial and Postmarketing Safety of Onasemnogene Abeparvovec Therapy. Drug Saf. *44*, 1109–1119. https://doi.org/10.1007/s40264-021-01107-6.

50. Strauss, K.A., Farrar, M.A., Muntoni, F., Saito, K., Mendell, J.R., Servais, L., McMillan, H.J., Finkel, R.S., Swoboda, K.J., Kwon, J.M., et al. (2022). Onasemnogene abeparvovec for presymptomatic infants with two copies of SMN2 at risk for spinal muscular atrophy type 1: the Phase III SPR1NT trial. Nat. Med. *28*, 1381–1389. https://doi.org/10.1038/s41591-022-01866-4.

51. Zhang, H., Wan, Z., Li, X., Wang, B., Guan, J., Li, Y., Jin, X., Ma, X., and Liu, G. (2025). Adenine base editing rescues disrupted BCKDH function and reduces BCAAs toxic accumulation in maple syrup urine disease patient iPSC-hepatic organoids. Stem Cell Res. Ther. *16*, 512. https://doi.org/10.1186/s13287-025-04630-w.

52. Morton, D.H., Strauss, K.A., Robinson, D.L., Puffenberger, E.G., and Kelley, R.I. (2002). Diagnosis and treatment of maple syrup disease: a study of 36 patients. Pediatrics *109*, 999–1008.

53. Chuang, J.L., Wynn, R.M., Moss, C.C., Song, J.L., Li, J., Awad, N., Mandel, H., and Chuang, D.T. (2004). Structural and biochemical basis for novel mutations in homozygous Israeli maple syrup urine disease patients: a proposed mechanism for the thiamin-responsive phenotype. J. Biol. Chem. *279*, 17792–17800.

54. Wynn, R.M., Davie, J.R., Chuang, J.L., Cote, C.D., and Chuang, D.T. (1998). Impaired assembly of E1 decarboxylase of the branched-chain alpha-ketoacid dehydrogenase complex in type IA maple syrup urine disease. J. Biol. Chem. *273*, 13110–13118.

55. FDA (2026). Biomarker Qualification Program. https://www.fda.gov/drugs/drug-development-tool-ddt-qualification-programs/biomarker-qualification-program.

STAR★METHODS

KEY RESOURCES TABLE

REAGENT or RESOURCE	SOURCE	IDENTIFIER
Biological samples		
Human plasma samples (venous)	Clinic for Special Children	N/A
Dried blood spot (DBS) samples	Clinic for Special Children	N/A
Chemicals, peptides, and recombinant proteins		
Anamix Early Years (MSUD formula)	Nutricia North America	Product Code: 90168
L-Isoleucine (50 g)	Nutricia North America	Product Code: 0170V
L-Valine (50 g)	Nutricia North America	Product Code: 0140I
L-Leucine (50 g)	Nutricia North America	Product Code: 0150L
Critical commercial assays		
Quantitative amino acids by HPLC (Agilent system)	Clinic for Special Children (CLIA-certified)	N/A
Deposited data		
De-identified individual-level clinical data (MATCH cohort)	This paper; lead contact	N/A
Software and algorithms		
GraphPad Prism (version 10.5)	GraphPad Software (San Diego, CA)	RRID:SCR_002798; https://www.graphpad.com
R (version 4.5.1)	R Foundation for Statistical Computing (Vienna, Austria)	RRID:SCR_001905; https://www.r-project.org
Monte Carlo simulation code for power analyses	This paper; lead contact	https://doi.org/10.5281/zenodo.19335826
Other		
WHO-MGRS growth reference standards	World Health Organization	https://www.who.int/tools/child-growth-standards/standards/weight-for-age
WHO-MGRS motor milestone windows	World Health Organization	WHO MGRS Group[44]
Abbreviations: N/A, not applicable.		

EXPERIMENTAL MODEL AND STUDY PARTICIPANT DETAILS

Human subjects

The MSUD Age-matched Standard Treatment Cohort (MATCH) represents a convenience population of young children with classic MSUD receiving clinical care at the CSC (Gordonville, PA). All cases diagnosed and managed at CSC between 2019 and 2023 were screened for inclusion. Early diagnosis occurred either through newborn screening or through immediate postnatal testing based on known parental carrier status, reflecting a preemptive population genetics approach rather than disease severity. Participants had a confirmed biochemical and molecular diagnosis of classic MSUD within seven postnatal days. Exclusion criteria were applied uniformly and independent of clinical course.

Children were excluded if they received a substantial portion of their care outside CSC, were unable to adhere to the institution's treatment and monitoring protocols, or had a confounding medical condition likely to alter clinical course or amino acid homeostasis; exclusions were unrelated to disease severity. The observation window extended from birth through liver transplantation or death, whichever occurred first.

The cohort comprised eight female and three male infants, as assigned by the attending physician at birth. Gestational age, birth weight, age at diagnosis, and duration of follow-up are summarized in Table 1. All participants were otherwise healthy at birth; none had immunodeficiency or clinically significant comorbidities. Participants received standard childhood immunizations in accordance with the CSC treatment protocol (Table S5), with vaccinations held during periods of illness or hyperleucinemia. No participant had received gene therapy, investigational treatment, or liver transplantation prior to enrollment. Dietary management, amino acid monitoring, and crisis management protocols are described in method details.

CellPress
OPEN ACCESS

The study was approved by the Penn Medicine–Lancaster General Hospital Institutional Review Board (Protocol #2008–095-CSC). Written informed consent for research participation was obtained from parents or legal guardians. Parents of the child depicted in Figure 1A signed a photo consent permitting reproduction of the exact image shown.

METHOD DETAILS

Clinical care and follow-up

All participants were followed longitudinally at CSC under a standardized clinical protocol aligned with published consensus standards (Table S5). During the first year of life, patients were typically evaluated by a physician monthly, with visit frequency decreasing to every 2–3 months thereafter. At each clinic visit, clinicians obtained a medical history, performed a physical examination, reviewed dietary records and laboratory results, and documented concomitant treatments and adverse events. Growth and motor development were benchmarked against WHO-MGRS standards,[44] and growth curves were generated from clinic-recorded measurements.

Dietary management

Dietary therapy consisted of intact protein sources (including expressed human milk, commercial infant formulas, and weighed table foods), BCAA-free formula (Nutricia North America, Montreal), and variable quantities of liquid isoleucine, valine, and occasionally leucine (10 mg/mL solutions prepared in distilled water). All dietary components were measured using standardized units (milligrams, grams, milliliters, or ounces) with measuring cups or digital gram scales.

Diet prescriptions were adjusted frequently to (1) maintain plasma leucine concentrations within the reference range observed in healthy pediatric controls (mean 119 ± 38 μM, range 62–200), (2) prevent iatrogenic BCAA deficiencies, and (3) preserve physiologic leucine/isoleucine and valine/leucine ratios (Table S5).

Amino acid monitoring

Quantitative amino acids (AAQs) were measured by high-performance liquid chromatography using plasma samples collected during clinic visits or dried blood spot (DBS) samples submitted from home. Families were instructed to submit AAQ samples once or twice weekly between clinic visits.

AAQ results were typically communicated to families within 0.5 h for plasma samples and within 32 h for DBS samples, often accompanied by dietary review and modification. Because reference ranges for BCAAs do not differ between plasma and DBS matrices,[39,40] data from both sources were pooled for analysis.

DBS samples were generally collected in the morning before or after the first meal, whereas plasma samples were obtained during daytime clinic visits or inpatient admissions. Blood collection was not timed to feeding or fasting state; therefore, aggregated AAQ data reflect values obtained across a range of physiologic conditions.

Management of intercurrent illness

Episodic illnesses were managed at home or in the hospital depending on clinical severity. For non-encephalopathic children with moderate hyperleucinemia, home management consisted of calorie-dense "sick-day" formulas devoid of intact protein and enriched with BCAA-free amino acids. Dietary adjustments were guided by AAQ monitoring every 1–3 days until blood leucine concentrations normalized.

Hospitalization was prompted by encephalopathy, significant catabolic illness, gastrointestinal intolerance, or persistent hyperleucinemia. Inpatient management followed a standardized protocol including BCAA-free parenteral nutrition, intravenous isoleucine and valine supplementation, continuous intravenous insulin, judicious use of hyperosmolar therapy, and laboratory monitoring every six hours (Table S5).[1,4,39]

QUANTIFICATION AND STATISTICAL ANALYSIS

Descriptive and comparative analyses

Statistical analyses were performed using Prism 10.5 (GraphPad). Descriptive statistics are reported as mean ± standard deviation (SD), median with 25th–75th percentile interquartile range (IQR), absolute range, and coefficient of variation (CV). Growth parameters and motor milestones were compared with WHO-MGRS reference standards.[44] Time-to-event analyses for sitting and walking milestones were compared with those of healthy Amish and Mennonite control children (n = 18) using Mantel–Cox log rank tests. The proportional hazards assumption for the Mantel-Cox log rank test was verified by visual inspection of Kaplan-Meier curves. The curves showed similar developmental trajectories between the MATCH cohort and healthy controls, with no sustained divergence, supporting the use of the log rank test for this comparison.

Amino acid concentrations in MATCH participants were compared with pediatric control data using unpaired t-tests with Welch's correction. Plasma BCAA concentration ratios (leucine/isoleucine and valine/leucine) were log10-transformed before analysis. Associations between variables were evaluated using nonparametric Spearman correlation coefficients (r_s). For analyses involving repeated measurements, 'N' denotes the number of subjects and 'n' denotes the number of samples. For comparisons between

MATCH participants and pediatric controls in Figure 4, subject-level mean values were used to ensure independence of observations and avoid pseudoreplication arising from unequal sampling frequency.

Estimand framework and power simulations

Key outcome measures were framed as estimands in accordance with the ICH E9(R1) addendum, *Estimand and Sensitivity Analysis in Clinical Trials*.[35] Estimands specify the clinical question of interest and define treatment effects using six components, enabling alignment between outcome definition and statistical inference.[36]

Given the anticipated use of MATCH as an external comparator group for small, single-arm trials, conventional power methods were not appropriate. Power analyses therefore evaluated whether a hypothetical treatment group of 11 participants could achieve $\geq$90% power to detect clinically meaningful differences relative to MATCH controls and identified the minimum detectable effect size for each outcome. A 1:1 allocation ($N = 11$ per arm) was assumed to reflect feasible recruitment constraints in an ultra-rare pediatric disease and to provide a conservative, realistic comparison framework. Monte Carlo simulations were performed in R (version 4.5.1). Control group means and standard deviations were derived from subject-level averages in MATCH, while treatment group variances were informed by preclinical studies[28] and comparable clinical cohorts.[1,15]

Normality assumptions for these simulations were assessed via Shapiro-Wilk testing and found adequate for parametric modeling of subject-level means. To account for parameter uncertainty and potential non-normality in metabolic or rate data, we performed sensitivity analyses by varying hypothesized treatment group standard deviations by ±20%, which confirmed the stability of the reported power estimates and minimum detectable effects.

For a range of plausible treatment means, 10,000 simulated trials were generated assuming normally distributed outcomes and equal allocation (treatment:control). Each simulated dataset was analyzed using Welch's two-sample *t* test with statistical significance defined as two-sided $p \leq 0.025$, consistent with FDA recommendations. This threshold was prespecified to reflect FDA guidance, which indicates that a single adequate and well-controlled study intended to support substantial evidence of effectiveness should meet a more stringent statistical standard than the traditional two-trial paradigm (two-sided $\alpha = 0.050$).[45] Empirical power was calculated as the proportion of simulations achieving $p \leq 0.025$, and the smallest treatment mean achieving $\geq$90% power was recorded. Estimands and their components are summarized in Table S2.

Cell Reports Medicine, Volume *7*

Supplemental information

Trial-ready external controls for gene therapy: The MATCH cohort in maple syrup urine disease

Karlla W. Brigatti, Ashlin Rodrigues, Erin Sweigert, Joelle Williamson, Alanna Koehler, Grace Loudon Meier, Laura E. Poskitt, Vincent J. Carson, Donna Robinson, and Kevin A. Strauss

Table S1. Six clinically relevant outcome measures to support MSUD gene therapy trials (Related to Figures 2, 3, and 4)

Outcome Measure	Clinical Relevance
**Crisis Management Days**	Each CMD is a proxy for life-threatening metabolic encephalopathy (*5*). Once elevated, neurotoxic concentrations of BCAAs and BCKAs can only be normalized by intensive anabolic therapy (*56*) and/or hemodialysis (*57*), both of which require advanced medical resources and expertise (*39*).
**Proportional Intact Protein Equivalent**	PIPE represents the proportion (%) of total ingested protein from intact (natural) as compared to BCAA-free sources and quantifies how prescription diet differs from normal unrestricted food intake. A PIPE of 100% means 'dietary freedom.'
**Leucine Tolerance**	Leucine tolerance represents the sum of leucine accretion in tissues plus its insensible loss in skin, hair, nails, and excreta *(56, 58)*. It varies directly with growth rate *(1)*.
**Alloisoleucine**	Alloisoleucine is a pathognomonic marker of BCKDH deficiency (*40, 42*). In a large retrospective study of 184 MSUD patients, its blood concentration was 190±117 µM on diet, 4±8 µM after liver transplantation, and undetectable in control subjects (*1*).
**Branched-chain Amino Acids**	Leucine and 2-ketoisocaproic acid are the principal neurotoxins in MSUD and their concentrations in blood correlate with short- and long-term neurological impairments (*23, 59-62*). Following liver transplantation, BCAAs remain modestly elevated but stable in the face of unrestricted daily protein ingestion (*1, 14, 15*).
**BCAA Concentration Ratios**	The intact BCKDH complex maintains concentration relationships among the three BCAAs such that plasma ratios (mol:mol) of leucine to isoleucine (Leu/Ile) and valine to leucine (Val/Leu) are between 1.5 and 2.1 under most conditions (*1*). In contrast, Leu/Ile and Val/Leu ratios vary by orders magnitude in patients with classic MSUD (*1*).

Abbreviations: BCAA, branched-chain amino acid; BCKA, branched-chain 2-ketoacids; BCKDH, branched-chain 2-ketoacid dehydrogenase; CMD, crisis management day; Ile, isoleucine; Leu, leucine; MSUD, maple syrup urine disease; PIPE, proportional intact protein equivalent; Val, valine.

Table S2. Estimand framework for MSUD clinical trials (Related to Figure 5)

	PIPE (%)	CMDs (days/year)	Alloisoleucine (µM)
**Population**	Children from birth to age 24 months with biallelic pathogenic variants in *BCKDHA* or *BCKDHB*, a clinical-biochemical phenotype consistent with MSUD, and no concomitant diagnoses that might confound interpretation of efficacy data.		
**Intervention**	Systemic single-gene or dual-gene replacement or editing of *BCKDHA* and/or *BCKDHB.*		
**Comparison Treatment**	Standard BCAA-restricted dietary therapy paired with inpatient/outpatient crisis management protocols (i.e., current standard of care).		
**Outcome (Meaningful)**	≥50% increase in the proportion of ingested protein from intact ('natural') sources, measured as % of total protein intake.	≥50% reduction in CMDs for management of metabolic instability, counted in days per patient per year.	≥30% reduction of the average alloisoleucine concentration in plasma and/or DBS samples (measured in µM).
**Summary Measure**	Average (SD) age-adjusted PIPE measured between 40 and 52 weeks post-intervention.	Average (SD) frequency of CMDs counted from 12 to 52 weeks post-intervention.	Average (SD) blood alloisoleucine measured between 40 and 52 weeks post-intervention.
**Intercurrent Events**	Patients who die or receive a liver transplant within 40 weeks of intervention are excluded from outcome analysis; i.e. the efficacy cohort includes only children "*while on treatment, while alive*."		

Abbreviations: CMDs, crisis management days; DBS, dried filter paper blood spot; PIPE, proportional intact protein equivalent; SD, one standard deviation.

Table S3. Dietary indices of classic maple syrup urine disease (N=11, n=1353; Related to Figure 3)

	Proportional Intact Protein Equivalent, %					Dietary Leucine Tolerance, mg/kg•day				
Age Range	n[a]	Mean (SD)	Median (IQR)	Range	CV	n[a]	Mean (SD)	Median (IQR)	Range	CV
**Birth to <1.5 mo**	*57*	28.4 (5.9)	30 (24-33)	17-40	21%	*21*	74 (19)	71 (64-85)	35-107	25%
**1.5 to <3 mo**	*83*	24.1 (5.4)	24 (19-28)	15-36	22%	*29*	53 (13)	52 (44-63)	24-77	25%
**3 to <6 mo**	*206*	19.0 (3.7)	19 (17-22)	11-29	20%	*93*	33 (8)	33 (26-37)	18-58	25%
**6 to <9 mo**	*193*	15.9 (4.0)	16 (13-19)	6-27	25%	*85*	24 (7)	22 (19-27)	8-40	28%
**9 to <12 mo**	*197*	14.8 (3.1)	15 (13-16)	6-23	21%	*76*	19 (3)	19 (18-21)	12-30	17%
**12 to <18 mo**	*337*	13.2 (4.4)	12 (10-16)	5-28	33%	*123*	16 (6)	15 (13-18)	5-38	35%
**18 to <24 mo**	*193*	12.2 (3.7)	12 (9-15)	5-22	30%	*85*	12 (3)	11 (9-13)	5-19	26%
**24 to 36 mo**	*87*	11.1 (3.1)	11 (8-14)	6-17	28%	*45*	11 (2)	10 (9-12)	8-16	21%
**12 to 36 mo[b]**	*617*	12.3 (2.5)	13 (11-16)	5-28	32%	*253*	14 (5)	13 (10-16)	5-38	36%

Abbreviations: CV, coefficient of variation; IQR, 25th to 75th interquartile range; mo, months; SD, one standard deviation.

[a] For each participant, individual dietary assessments were spaced at least three days apart. Median sampling frequency was every 5 days (IQR 4-7 days; range: 4-9 days). Leucine tolerance was calculated only on dates that correspond with a clinic visit (i.e., reliable weight measurement), meaning fewer values per age category; **[b]** Anticipating future gene therapy trials, we aggregated ages 12-36 months into a single category (*bottom row*) to perform power analysis for the PIPE estimand.

Table S4. Biomarkers to support clinical trials: MSUD patients (N=11) versus pediatric control subjects (N=51; Related to Figure 4)

	**MSUD** (N=11, n=1729)				**Control** (N=51, n=51)				
	Mean (SD)	Median (IQR)	Range	CV	Mean (SD)	Median (IQR)	Range	CV	p value[b]
**Alloisoleucine, µM**	190 (120)[a]	162 (107-245)	0-893	63%	nd	-		-	na
**Leucine, µM**	180 (151)	137 (85-227)	15-2172	76%	119 (38)	112 (86-151)	62-200	32%	*<0.0001*
**Isoleucine, µM**	246 (187)	198 (126-303)	8-1620	84%	65 (25)	61 (46-84)	26-121	38%	*<0.0001*
**Valine, µM**	609 (246)	573 (447-730)	55-2378	40%	208 (61)	208 (150-263)	118-335	30%	*<0.0001*
**Leu/Ile ratio, mol:mol**	1.14 (1.56)	0.81 (0.42-1.39)	0.01-28.1	136%	1.91 (0.37)	1.83 (1.64-2.13)	1.27-3.41	20%	*<0.0001*
**Val/Leu ratio, mol:mol**	5.95 (6.49)	3.83 (2.25-7.20)	0.27-62.86	109%	1.78 (0.28)	1.77 (1.63-2.00)	1.13-2.49	16%	*<0.0001*

Abbreviations: CV, coefficient of variation; IQR25-75, 25th to 75th interquartile range; N, number of individual subjects; n, number of samples; na, not applicable; nd, not detected; SD, one standard deviation.

[a] For estimand power analysis, we used an average alloisoleucine value of 183±50 µM, which represents the 753 samples obtained between ages 12 and 36 months;
[b] Unpaired, two-tailed t-test with Welch's correction (does not assume equal SDs).

Table S5: Standardized MSUD treatment and monitoring protocol (Clinic for Special Children; Related to Figure 1)

Clinical Context	Treatment Goals	Interventions	Monitoring Principles
**Newborn period**	1. Prevent crises<br>2. Normal plasma BCAAs<br>3. Normal growth	***Asymptomatic Outpatient Management***<br>▪ Only BCAA-free MSUD formula for 24-48 hours<br>▪ Leucine normalizes, start 60-70mg/kg-day leucine from intact source + BCAA-free MSUD formula[a]<br>▪ Supplement 5mg/kg-day L-isoleucine and 20mg/kg-day L-valine; titrate to physiologic ratios<br>▪ Feeding frequency: every 2-3 hours<br>▪ Consider thiamine trial (100-1000 mg).<br>***Symptomatic Infant***<br>▪ Admit for inpatient management	***Plasma Amino Acid Analysis:***<br>▪ Quantitative amino acids (AAQ) at birth or diagnosis<br>▪ Recheck AAQ after 24-48 hour 'washout' with BCAA-free formula only (no intact protein)<br>▪ Then, twice weekly and more frequently as needed
**Outpatient 'Well Day'**	1. Normal growth<br>2. Normal development<br>3. Prevent nutrient deficiencies	***Diet and Monitoring***<br>▪ Age appropriate DRIs for calories and nutrients<br>▪ Total protein: 2-3.5g/ kg-day[b]<br>▪ Intact protein according to leucine tolerance<br>▪ Titrate L-isoleucine (0 -30mg/ kg-day) and L-valine (5-20mg/ kg-day)[c] to physiologic ratios<br>▪ Formula concentration: 0.7-1.0 kcal per mL (20-30 kcal per oz) as tolerated<br>▪ Feeding schedule: *ad lib* from 24-hour volume<br>▪ Standard immunizations[d]<br>▪ Avoid and treat catabolic stressors[e]	***Outpatient Visit Schedule:***<br>▪ Birth to 1 year: every month<br>▪ 1 to 2 years: every 2-4 months<br>▪ 2 years+: every 3-6 months<br>***Plasma Amino Acid Analysis:***<br>▪ Birth to 1 year: twice weekly<br>▪ 12-24 months: weekly<br>▪ 2 years+: every 1-2 weeks<br>▪ Goal: leucine 114 – 190 μmol/L<br>▪ BCAA stoichiometry leucine : isoleucine : valine ratio 2:1:4
**Outpatient 'Sick Day'**	1. Reverse catabolism<br>2. Promote anabolism<br>3. Treat precipitants<br>*4. Hospitalize as indicated*	***Diet and Monitoring***<br>▪ Restrict leucine intake for at least 24 hours<br>▪ BCAA-free protein ≥2-3.5g/ kg-day[b]<br>▪ Caloric intake ≥ resting energy expenditure[g]<br>▪ Formal intake every 1-3 hours, *even overnight*<br>▪ Increase L-isoleucine (30-60mg/kg-day) and L-valine (30-60mg/kg-day) supplementation<br>▪ Low threshold for antimicrobials<br>▪ Antipyretics, antiemetics, and supportive care	***Surveillance Measures***<br>▪ Plasma AAQ daily: target leucine 114-190 μmol/L<br>▪ Office visits to identify treatable illnesses<br>▪ Maintain daily contact with patient/family<br>***Admit to Inpatient for the Following:***<br>▪ Leucine ≥ 760 μmol/L or rapidly increasing<br>▪ Significant encephalopathy or signs of cerebral edema<br>▪ Vomiting, diarrhea, dehydration, or poor oral intake<br>▪ Physical trauma or surgical indication

[a] We use a 24-hour batch consisting of: intact protein source (formula or breastmilk) combined with BCAA-free formula, isoleucine, and valine. Mix additional batch as needed.
[b] Typically 3-3.5g/kg-day for infants < 6 months, 2.5-3g/kg-day for infants 6-12 months, and 1.5-2.5g/kg-day for toddlers.
[c] High relative affinity of leucine for blood-barrier transport compared to valine predisposes to cerebral valine deficiency, which correlates with poor neurocognitive outcomes, making continuous valine supplementation especially important [Muelly 2013]. [f] Higher valine levels (340-680 **μmol**/L) are well-tolerated and may be preferable to avoid cerebral valine deficiency.
[d] Hold immunization when patient is ill or has hyperleucinemia.
[e] Glucocorticoids contraindicated; they promote catabolism and should be avoided except for life-threatening illness.
[g] Minimize physical activity to avoid additional energy expenditure.

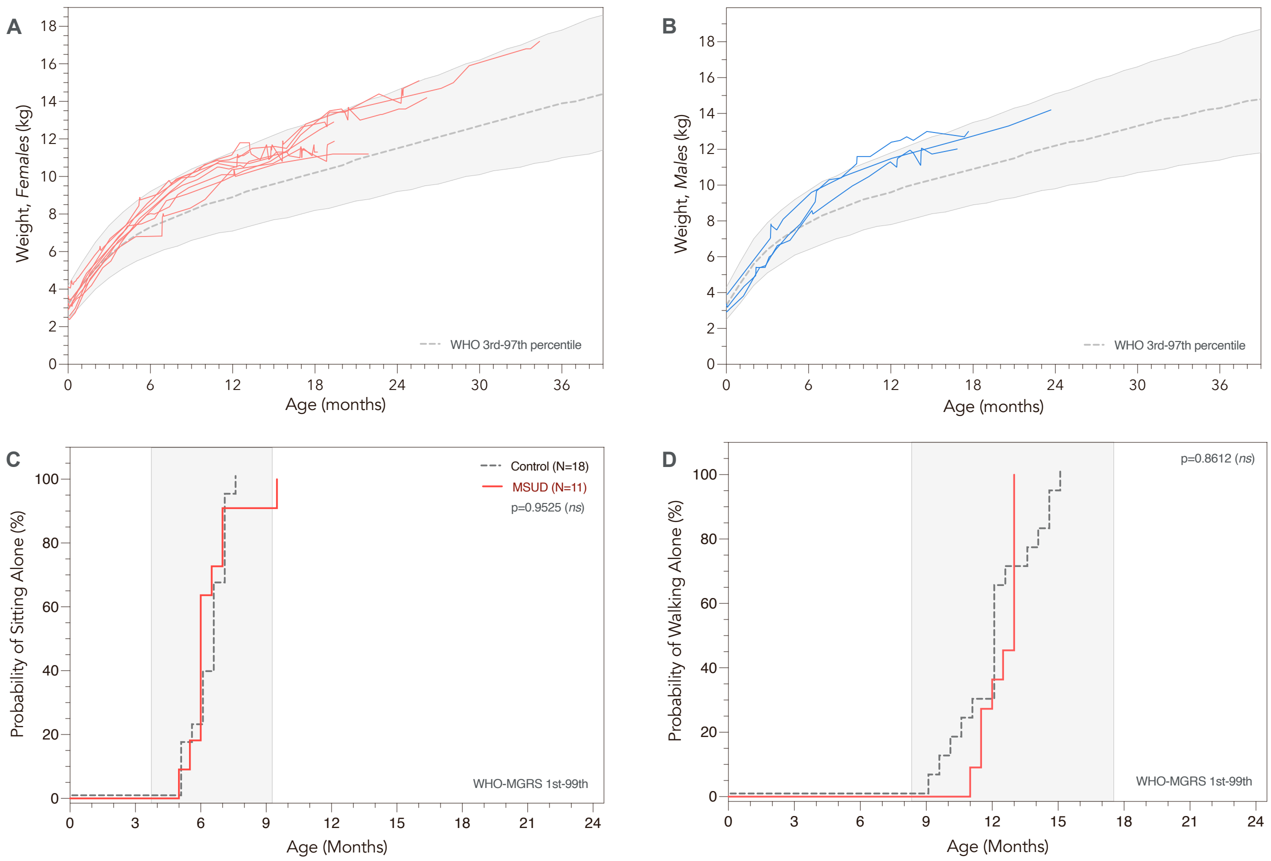

Figure S1. Growth and Early Motor Development. All participants of female (panel **A**, red lines) and male (panel **B**, blue lines) gender grew normally during the first 24 months of life according to World Health Organization (WHO) 3rd-97th percentile reference standards (gray shading). Length and head circumference were also normal (data not shown). All participants achieved independent sitting (panel **C**) and walking (panel **D**) milestones on time, comparable to those of 18 healthy, unrelated children, and within normal windows established by the WHO Multicentre Growth Reference Study (gray shading)[63]. Related to Table 1 and Figure 2.
